# Probiotic treatment induces sex-dependent neuroprotection and gut microbiome shifts after traumatic brain injury

**DOI:** 10.1186/s12974-025-03419-1

**Published:** 2025-04-20

**Authors:** Morgan Holcomb, Austin G. Marshall, Hannah Flinn, Mariana Lozano-Cavazos, Sirena Soriano, Fernando Gomez-Pinilla, Todd J. Treangen, Sonia Villapol

**Affiliations:** 1 Department of Neurosurgery and Center for Neuroregeneration, Houston Methodist Research Institute, Houston, TX USA; 2 Department of Computer Science, Rice University, Houston, TX USA; 3 Departments of Neurosurgery and Integrative Biology and Physiology, Brain Injury Research Center, University of California, Los Angeles, Los Angeles, CA USA; 4 Department of Bioengineering, Rice University, Houston, TX USA; 5 Ken Kennedy Institute, Rice University, Houston, TX USA; 6 Department of Neuroscience in Neurological Surgery, Weill Cornell Medical College, New York City, NY USA

**Keywords:** Microbiome, Microglia, Neuroinflammation, *Lactobacillus*, Gut-brain axis, Neuroprotection, Metabolites

## Abstract

**Background:**

Recent studies have highlighted the potential influence of gut dysbiosis on traumatic brain injury (TBI) outcomes. Alterations in the abundance and diversity of *Lactobacillus* species may affect immune dysregulation, neuroinflammatory responses, anxiety- and depressive-like behaviors, and neuroprotective mechanisms activated in response to TBI.

**Objective:**

This study aims to evaluate the protective and preventive effects of Pan-probiotic (PP) treatment on the inflammatory response during both the acute and chronic phases of TBI.

**Methods:**

Males and female mice underwent controlled cortical impact (CCI) injury or sham. They received a PP mixture in drinking water containing strains of *Lactobacillus plantarum*,* L. reuteri*,* L. helveticas*,* L. fermentum*,* L. rhamnosus*,* L. gasseri*, and *L. casei.* In the acute group, mice received PP or vehicle (VH) treatment for 7 weeks before TBI, continuing until 3 days post-injury (dpi). In the chronic group, treatment began 2 weeks before TBI and was extended through 35 dpi. The taxonomic microbiome profiles of fecal samples were evaluated using 16S rRNA V1-V3 sequencing analysis, and Short-chain fatty acids (SCFAs) were measured. Immunohistochemical, in situ hybridization, and histological analyses were performed to assess neuroinflammation post-TBI, while behavioral assessments were conducted to evaluate sensorimotor and cognitive functions.

**Results:**

Our findings suggest that a 7-week PP administration induces specific microbial changes, including increased abundance of beneficial bacteria such as *Lactobacillaceae*, *Limosilactobacillus*, and *Lactiplantibacillus*. PP treatment reduces lesion volume and cell death at 3 dpi, elevates SCFA levels at 35 dpi, and decreases microglial activation at both time points, particularly in males. Additionally, PP treatment improved motor recovery in males and alleviated depressive-like behaviors in females.

**Conclusion:**

Our findings indicate that PP administration modulates microbiome composition, reduces neuroinflammation, and improves motor deficits following TBI, with these effects being particularly pronounced in male mice.

**Supplementary Information:**

The online version contains supplementary material available at 10.1186/s12974-025-03419-1.

## Background

Traumatic brain injury (TBI) is a leading cause of death and disability worldwide [1], with long-term consequences that include an elevated risk of neurodegenerative disorders, such as Alzheimer’s and Parkinson’s diseases [2, 3]. Currently, there are no effective treatments for neuroprotection or the regeneration of damaged brain tissue in either the short- or long-term. The pathophysiology of TBI is heavily influenced by neuroinflammation, primarily driven by microglial cells [4], along with infiltrating macrophages, neutrophils, and leukocytes. These immune responses contribute to the secondary injury processes that affect cognitive and motor recovery [5]. Microglia, the resident immune cells of the central nervous system, are key players in the brain’s response to injury. Upon activation, they transition from a resting state to a reactive state [6], releasing pro-inflammatory cytokines [4]. Neuroinflammation triggered by TBI can also impact peripheral organs, such as the gut, by inducing systemic inflammation that compromises gut permeability and disrupts the gut microbiome, leading to dysbiosis. The gut microbiota, in turn, influences microglial maturation and activation [7], creating a bidirectional feedback loop that exacerbates both peripheral and central inflammation [8].

The presence or absence of specific symbiotic bacteria in the gut significantly influences the brain’s response to chronic inflammation and its capacity to recover from certain neuropathological conditions [9]. This connection is mediated through the bloodstream and vagus nerve signaling pathways [10], profoundly affecting mental health [11] and contributing to the development of neurological disorders [12]. The microbiome plays a vital role in preserving the integrity of the blood-brain barrier (BBB) [13], supporting myelin formation [14], and regulating microglial activation [15], all of which are critical for maintaining proper neurological function.

The gut microbiome plays a crucial role in brain function under healthy physiological conditions, influencing neurotransmission, neurogenesis, and glial activation [16] processes. This dynamic interaction, known as the gut-microbiome-brain axis, is essential for maintaining neurological health [17], contributing to the progression of neuropathological conditions [18]. Interestingly, individuals who survive TBI often develop gastrointestinal (GI) dysfunction [12]. Our previous animal [19] and clinical [20] studies have demonstrated a clear link between TBI and bacterial dysbiosis characterized by heightened systemic inflammation, impaired intestinal motility, and exacerbated neuroinflammation [21–23]. Disruptions in the gut microbiota are associated with poorer TBI outcomes, contributing to neurodegeneration [24] and negative impacts on brain recovery [25].

The *Lactobacillaceae* family play a vital role in maintaining gut health and are commonly found in the human GI system [26]. A decrease or absence of these beneficial microbes can amplify neuroinflammation and exacerbate neurocognitive impairments associated with TBI [27]. The gut microbiota produce neurotransmitters, microbial byproducts, and metabolites such as short-chain fatty acids (SCFAs), vital for CNS homeostasis. TBI has been linked to decreased levels of SCFAs, which negatively impact the healing process [28]. The benefits associated with the production of SCFAs, including butyrate, propionate, and acetate, are essential in reducing inflammation and oxidative stress [29], maintaining BBB integrity, stimulating neurogenesis, reducing neuroinflammation, and promoting microglial maturation [30, 31]. Additionally, probiotic treatments, which involve administering live beneficial microorganisms like *Lactobacillus* strains, have been shown to boost SCFA levels [32, 33], potentially offering significant health benefits to the host [34].

Despite previous findings, the role of *Lactobacillus* strains in modulating microglia activity and improving motor and cognitive functions through alterations in gut microbiota composition in a mouse model of TBI remains unexplored. In this study, we tested the hypothesis that administering a mixture of probiotics derived from *Lactobacillus* strains after TBI could mitigate gut dysbiosis and enhance motor function by reducing the neuroinflammatory response. Our results support the potential of *Lactobacillus spp.* as a therapeutic strategy for TBI management. Given probiotics’ well-established safety and tolerability, this approach offers a promising and readily translatable option for clinical applications.

## Materials and methods

### Mice

We used male and female C57BL/6 mice, aged 9–12 weeks and weighing 20–26 g, obtained from Jackson Laboratories (Bar Harbor, ME, US). The mice were housed in an environment with a 14-h light/10-h dark cycle at a stable temperature of 23 °C ± 2 °C and had continuous access to food and water. After arriving, the mice were given at least 3 days of acclimatization to adjust to their new surroundings before participating in experimental procedures. Both adult male and randomly cycling female C57BL/6 mice were divided into groups for the study. Animal studies were performed under the National Institute of Health guidelines. Institutional Animal Care approved all experiments and Use Committees at Houston Methodist Research Institute (Houston, TX).

### Traumatic brain injury model

Anesthesia was induced with 4–5% isoflurane and maintained at 1.5–2% with an oxygen flow rate of 1–1.5 L/min. Once anesthetized, each mouse was secured in a stereotaxic frame for the surgical procedure. A moderate-to-severe TBI was delivered to the left cortex, explicitly targeting the primary motor and somatosensory cortices, using a controlled cortical impact (CCI) injury system driven by an electromagnetic device (Leica StereoOne Impactor). The mouse’s body temperature was maintained at 37 °C throughout the surgical procedure using a heating pad. A midline incision was approximately 10 mm in length on the skull, and the skin and fascia were retracted to expose the underlying bone. A 4 mm craniotomy was performed on the left parietal bone to access the cortical surface. A 1.5 mm in diameter sterilized impactor tip was positioned on the exposed dura and set to deliver a CCI with a velocity of 3.25 m/s and a tissue deformation of 1.5 mm. Mice in the sham-operated group underwent identical procedures, including anesthesia with isoflurane and craniotomy, but without the CCI injury. Following the TBI procedure, the incision was sutured, and the mice were placed in a warmed recovery chamber to maintain normal body temperature during recovery. All animals were closely monitored for 4 h post-surgery and checked daily thereafter to ensure proper recovery.

### Preparation of bacterial cell suspension for oral feeding

The probiotic (PP) mixture consisted of various *Lactobacillus* strains obtained from the American Type Culture Collection (ATCC) (Manassas, VA, USA). The included strains were *Lactobacillus gasseri* (ATCC 33323), *Lactobacillus plantarum* (ATCC BAA-793), *Lactobacillus reuteri* (ATCC 23272), *Lactobacillus helveticus* (ATCC BAA-2840), *Lactobacillus fermentum* (ATCC 23271), *Lactobacillus rhamnosus* (ATCC BAA-2836), and *Lactobacillus casei* (ATCC BAA-2843). Following the supplier’s guidelines, the strains were cultured in De Man, Rogosa, and Sharpe (MRS) medium or broth (Becton Dickinson, Sparks, MD) and preserved as glycerol stocks at -80 °C. To initiate culture growth, 5 mL of pre-warmed MRS broth was inoculated with glycerol stocks and incubated at 37 °C in a 5% CO₂ environment for 2 h. Starter cultures were subsequently transferred to 1 L of MRS broth and incubated overnight under identical conditions until the cultures reached the logarithmic growth phase, confirmed by measuring optical density at 600 nm (OD₆₀₀). Bacterial cells were then harvested through centrifugation at 3000 × g and 4 °C, washed in cold PBS, and resuspended in 10% glycerol in PBS. The resulting suspensions were aliquoted into 1 mL vials and promptly frozen for future administration to mice. To ensure consistency and accuracy, bacterial viability and concentrations were verified through serial dilutions and colony-forming unit (CFU) enumeration on agar plates.

### Probiotic administration

**Fig. 1:**
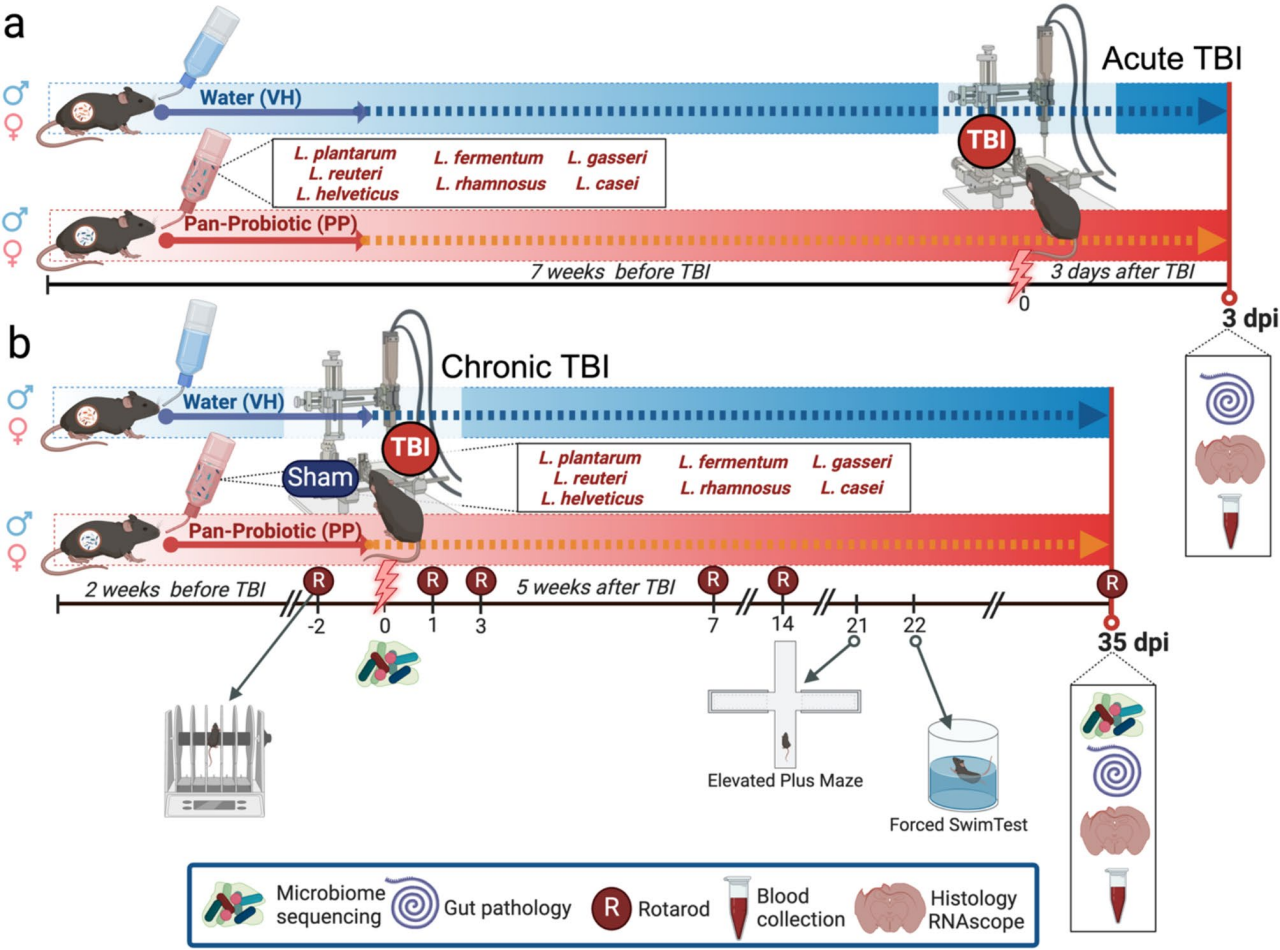
Schematic representation of the experimental design for acute and chronic TBI models with P Pan-Probiotic (PP) treatment. (**a**) Acute TBI Model: Mice received either water (VH) or a Pan-Probiotic (PP) cocktail containing *Lactobacillus plantarum*, *L. reuteri*, *L. helveticus*, *L. fermentum*, *L. rhamnosus*, *L. gasseri*, and *L. casei* via drinking water for 7 weeks prior to TBI induction. Acute assessments were performed 3 days post-injury (dpi), including microbiome sequencing, gut pathology analysis, histological examination, and blood collection. (**b**) Chronic TBI Model: Mice were pretreated with either water (VH) or the PP cocktail for 2 weeks before TBI or sham surgery. Post-TBI, treatment continued for 5 weeks, and outcomes were evaluated at multiple time points (7, 14, 21, and 35 dpi). Rotarod testing (R) was conducted to assess motor function, and behavioral tests, including the Elevated Plus Maze and Forced Swim Test, were performed to evaluate anxiety- and depressive-like behaviors. Additional analyses included microbiome sequencing, gut pathology, blood collection, histology, and RNAscope imaging at 35 dpi

Male and female C57BL/6 mice were divided into two groups and treated with PP in their drinking water or given water as a vehicle (VH): (1) for 7 weeks before TBI and continuing for 3 days post-injury (dpi) until euthanasia, and (2) for 2 weeks as a pretreatment, before receiving a TBI continuing for 5 more weeks until euthanasia at 35 dpi. For the acute TBI group (3 dpi), mice were distributed into (i) a TBI-VH group (injured VH-treated/water mice) and (ii) a TBI-PP group (injured PP-treated mice). For the chronic TBI group (35 dpi), mice were distributed into four different groups: (i) sham-VH group (sham-operated control, vehicle VH-treated/water), (ii) a sham-PP group (sham-operated with PP treatment), (iii) a TBI-VH group (injured VH-treated/water mice), and (iv) a TBI-PP group (injured PP-treated mice) (Fig. 1).

All mice were given a standard diet of either PP-treated or regular drinking water for VH groups. For euthanasia, mice were deeply anesthetized using isoflurane at either 3 dpi (acute injury) or 35 dpi (chronic injury) and underwent transcardiac perfusion with cold phosphate-buffered saline (PBS) followed by 4% paraformaldehyde. Brain tissues were then harvested for immunohistochemical analysis, and serum samples were collected via centrifugation of blood and stored at − 80 °C for SCFA analysis.

### Brain tissue preparation and quantification of lesion volume

Brains were fixed in 4% paraformaldehyde overnight. Following 24 h of fixation, they were cryopreserved in a 30% sucrose solution for 48 h. Coronal brain sections, 16 μm thick, were prepared using a cryostat (Epredia Cryostar NX50, Fisher Scientific, Waltham, MA). The sectioning process was done in intervals of 0.5 mm throughout the brain, beginning 1.56 mm anterior from the bregma landmark to ensure a consistent and thorough examination of the affected areas for subsequent histochemical analysis. An average of 10–12 brain sections, equally spaced from 0 to -2.70 mm relative to the bregma, were selected for cresyl violet staining to target the area affected by injury. Sections were placed on gelatin-coated glass slides (SuperFrost Plus, Thermo Fisher Scientific, IL) and submerged in 0.5% cresyl violet solution (Sigma-Aldrich, St. Louis, MO), prepared in distilled water and filtered. Following staining, the slides underwent a dehydration process in graded ethanol solutions (100%, 95%, 70%, and 50%) for 2 min each and were then cleared in xylene twice, each for 2 min. The sections were then covered using a Permount mounting medium (Thermo Fisher Scientific) for preservation. The lesion area was quantified by examining every 16th section throughout the full range of the lesion. The area of the ipsilateral hemisphere was measured for each corresponding section. Lesion volume was calculated by multiplying the total lesion area by the interval between sections. To determine the percentage of lesion volume, the lesion area was divided by the total area of the ipsilateral hemisphere, as previously described [35–38].

### Serum SCFA analysis

SCFAs were analyzed by derivatization procedure. 40 µL of serum was added to 40 µL of acetonitrile, vortexed, and centrifuged. 40 µL of the supernatant, 20 µL of 200 mM 12C6- 3-Nitrophenylhydrazine (3NPH), and 120 mM 1-Ethyl-3-(3-dimethyl aminopropyl) carbodiimide (EDC) were combined. 20 µL of hydrochloric acid was added and incubated for 30 min at 40 °C. The resulting mixture was cooled and made up to 1.91 mL with 10% aqueous acetonitrile. 5 µL of the sample was injected into Liquid Chromatography-Tandem Mass Spectrometry (LC/MS/MS). SCFAs were separated using mobile phases: 0.1% formic acid in water (mobile phase A) and 0.1% formic acid in acetonitrile (mobile phase B). Separation of metabolites was performed on Acquity UPLC HSS T3 1.8 μm (2.1 × 100mM). The SCFA were measured in ESI negative mode using a 6495 triple quadrupole mass spectrometer (Agilent Technologies, Santa Clara, CA) coupled to an HPLC system (Agilent Technologies, Santa Clara, CA) with multiple reaction monitoring (MRM). The acquired data was analyzed using Agilent Mass Hunter quantitative software (Agilent Technologies, Santa Clara, CA).

### Immunofluorescence analysis and cell death assay

Serial free-floating brain sections, 16 μm thick, were prepared at the level of the dorsal hippocampus for immunohistochemical analysis. The sections were processed using immunohistochemistry protocols, including three consecutive 5-min washes in PBS containing 0.5% Triton X-100 (PBS-T). Sections were then treated with 5% normal goat serum (NGS) in PBS-T to block nonspecific binding for 1 h at room temperature. Overnight incubation at 4 °C followed, using 3% NGS in PBS-T with primary antibodies targeting anti-rabbit Iba-1 (Wako, #019-19741) at a dilution of 1:500 for labeling microglia/macrophages and anti-rat F4/80 (R&D Systems, #MAB5580) at a dilution of 1:200. The following day the sections were washed 3 times for 5 min each in PBS-T and incubated with the corresponding secondary antibodies (all 1:1000, Invitrogen), for 2 h at room temperature. The sections were then rinsed with PBS three times for 5 min each and incubated in PBS with DAPI solution (1:50,000, Sigma-Aldrich, St. Louis, MO) for counterstained nuclei. The sections were rinsed with distilled water and covered with Fluoro-Gel with Tris Buffer mounting medium (Electron Microscopy Sciences, Hatfield, PA). Semiquantitative image analysis of the staining in the cortical regions was performed using Image J software, as previously described [37]. To assess cell death, brain sections were processed for DNA strand breaks using Terminal deoxynucleotidyl transferase dUTP nick end labeling (TUNEL) from Fluorescence In Situ Cell Death Detection kit (Roche Diagnostic, Indianapolis, IN) according to the manufacturer’s instructions.

### Image acquisition and sholl analysis of microglia morphology

To analyze the microglia morphology in the injured brains after 3 dpi, we used a Leica DMi8 confocal microscope to acquire 40X magnification z-stack images of the total thickness of 16 μm-thick brain sections from the injured cortex that were immunostained for Iba-1 (*n* = 4 per group). Z-stacks were collected at 2048 × 2048 resolution with 3 frame averages for each color channel and were exported as.TIF files. After importing the Z-stacks, we performed Sholl analysis of microglial morphology using the Neurolucida Explorer (MBF Bioscience) setting increments of 2 μm with a starting radius of 2 μm, and the branches were detected using the user-guided tree tracing. When examining microglial processes in detail using Sholl analysis, the number of intersections and the average length of processes were quantified across different distances from the cell body. We combined intersection data from each microglia in a region into single profile plots, enabling comparison of the overall morphology between cortical microglia in the injured brains treated with VH or PP.

### Fluorescent in situ hybridization with immunohistochemical labeling

Coronal brain sections were adhered to gelatin-coated glass slides (Superfrost Plus, Thermo Fisher Scientific) and preserved at − 80 °C until use. Fluorescent in situ hybridization (FISH) was performed as per the manufacturer’s instructions using RNAscope^®^ Technology 2.0 Red Fluorescent kit (Advanced Cell Diagnostics (ACD), Hayward, CA, US) as previously described [37, 39]. Brain tissue sections were dehydrated using an ethanol series of 50%, 70%, and two times 100% for 5 min. Subsequently they were boiled for 10 min with pretreatment 2 solution (citrate buffer). Lastly, the slides were incubated with pretreatment 3 solution (protease buffer) for 30 min before hybridization. For hybridization, sections were incubated at 40 °C for 2 h with specific target probes: Mus musculus Arg1 (Cat. No. 403431, ACD), separately with Mus musculus TNF-α (Cat. No.311081, ACD), and with Mus musculus Tgfb1 (Cat. No. 407751. ACD). In addition, the negative (Cat. No. 310043, ACD) and positive (Cat. No. 313911, ACD) control probes were applied and allowed to hybridize for 2 h at 40 °C. The amplification steps were performed according to the manufacturer’s directions. After FISH, the slides were co-stained with anti-rabbit Iba-1 as previously described.

### Quantitative analysis

For quantitative analysis of immunolabeled sections, we implemented unbiased, standardized sampling techniques to measure tissue areas corresponding to the injured cortex showing positive immunoreactivity, as we previously described [36, 37]. For proportional area measurements, the microglia/macrophages Iba-1-immunoreactivity was reported as the proportional area of tissue occupied by immunohistochemical stained cellular profiles within a defined target area. Thresholded images converted to 8-bit grayscale were made using ImageJ (NIH, Bethesda, MD). The thresholding function was then used to set a black-and-white threshold corresponding to the imaged field, with the averaged background subtracted. Once a threshold was set, the “*Analyze Particles*” function was used to sum up the total area of positive staining and calculate the fraction of the total positive area for the stain as previously described [37]. To quantify the number of TUNEL, Iba-1, and F4/80 + cells in the injured cortex, an average of four coronal sections from the lesion epicenter (− 1.34 to − 2.30 mm from Bregma) were counted and imaged for each animal. Double co-localization of TGF-β, Arg1, and TNF-α mRNA expression with Iba-1 staining was evaluated using *z*-stack acquisitions with a confocal microscope.

### Collection and extraction of fecal samples for DNA analysis

Fresh stool pellets were aseptically collected and placed in sterile tubes, immediately snap-frozen, and subsequently stored at − 80 °C for preservation. Genomic bacterial DNA was extracted from these frozen stool samples utilizing the QIAamp PowerFecal Pro DNA Kit (Qiagen, Germantown, MD). To facilitate DNA extraction, bead beating was carried out in three cycles, each lasting one min, at a speed of 6.5 m/s. There was a 5 min rest period between each cycle. This mechanical disruption was performed using a FastPrep-24 system (MP Biomedicals, Irvine, CA). The DNA isolation proceeded per the manufacturer’s instructions, following the bead-beating process. The concentration of the extracted genomic DNA was subsequently quantified using a DS-11 Series Spectrophotometer/Fluorometer (DeNovix, Wilmington, DE).

### Sequencing of 16S rRNA V1-V3 regions

The 16S ribosomal RNA gene region V1 to V3 was targeted for microbiome characterization. The primers used for amplification contain adapters for MiSeq sequencing and single-index barcodes so that the PCR products may be pooled and sequenced directly, targeting at least 10,000 reads per sample [40]. Primers used for the 16S V1-V3 amplification were 27 F (AGAGTTTGATYMTGGCTCAG, where Y = C (90%) or T (10%); M = A (30%) or C (70%)) and 534R (ATTACCGCGGCKGCTGG, where K = G (10%) or T (90%)) [41]. Amplicons were generated using primers corresponding to the variable regions, and the PCR products were purified. Subsequently, sequencing libraries for the V1-V3 target were constructed following the instructions provided by the Illumina MiSeq system with end products of 300 bp paired-end libraries.

### Amplicon sequence analysis pipeline

Raw data files in binary base call (BCL) format were converted into FASTQs and demultiplexed based on the single-index barcodes using the Illumina ‘bcl2fastq’ software. Demultiplexed read pairs underwent an initial quality filtering using bbduk.sh (BBMap version 38.82), removing Illumina adapters, PhiX reads and reads with a Phred quality score below 15 and length below 100 bp after trimming. 16S V1-V3 quality-controlled reads were then merged using bbmerge.sh (BBMap version 38.82), with merge parameters optimized for the 16S V1-V3 amplicon type (vstrict = t qtrim = t trimq = 15). Further processing was performed using nf-core/ampliseq version 2.8.0 of the nf-core collection of workflows, utilizing reproducible software environments from the Bioconda and Biocontainers projects [42–45]. Data quality was evaluated with FastQC (version 0.12.1) and summarized with MultiQC (version 1.18) [46]. Sequences were processed sample-wise (independent) with DADA2 (version 1.28) to eliminate PhiX contamination, trim reads (forward reads at 275 bp and reverse reads at 265 bp; reads shorter than this were discarded), discard reads with > 2 expected errors, to correct errors, to merge read pairs, and to remove PCR chimeras. Ultimately, 3880 amplicon sequencing variants (ASVs) were obtained across all samples [47]. Between 29.81% and 44.06% of reads per sample (average 36.8%) were retained. The ASV count table contained 2,337,653 counts, at least 9201 and 34,821 per sample (average 19980). Taxonomic classification was performed by DADA2 and the database ‘Silva 138.1 prokaryotic SSU’ [48]. ASV sequences, abundance, and DADA2 taxonomic assignments were loaded into QIIME2 (version 2023.7) [49]. Within QIIME2, the final microbial community data were collected into an.rds file found within the phyloseq (version 1.44) folder of the nf-core ampliseq output [50]. The resulting phyloseq data frame object was merged with the phylogenetic tree calculated within QIIME2. The phyloseq object was used in the creation of alpha and beta diversity plots, PERMANOVA calculations with vegan::adonis2 (version 2.6-5), relative abundance bar plots and centered log-ratio (CLR) transformation with microViz (version 0.12.5), bioinformatic analysis was completed using nextflow (version 23.0.1) and R (version 4.3.2) scripts, and differentially abundant taxa calculations were performed using ANCOMBC2 [51].

### Western blot analysis

Serum was collected by allowing the blood to clot at room temperature for 30 min, followed by centrifugation at 4000 revolutions per minute (rpm) and 4 °C for 20 min. The serum was then stored at − 80 °C. For analysis, serum samples were diluted 1:5 with Laemmli sample buffer (Bio-Rad Laboratories, Hercules, CA) and heated at 100 °C for 10 min. The samples were then loaded onto 12% Mini-PROTEAN TGX Stain-Free gels (Bio-Rad Laboratories, Hercules, CA) for protein separation. Following electrophoresis, proteins were transferred to nitrocellulose membranes (Bio-Rad Laboratories, Hercules, CA). The membranes underwent blocking with 5% w/v skim milk powder in PBS-Tween 20 (PBS-Tw) for 1 h at room temperature. They were then incubated overnight at 4 °C with a goat anti-SAA primary antibody (1:500; AF2948, R&D Systems, Minneapolis, MN). This was followed by a 1 h room temperature incubation with a horseradish peroxidase-conjugated rabbit anti-goat secondary antibody (1:3000; Thermo Fisher Scientific, Waltham, MA). The membranes were developed using Clarity Western ECL (Bio-Rad Laboratories, Hercules, CA) and imaged with a ChemiDoc MP system (Bio-Rad Laboratories, Hercules, CA). Densitometry quantification and chemiluminescence imaging were carried out using ImageLab 6.0.1 software (Bio-Rad Laboratories, Hercules, CA).

### Behavioral tests

Investigators blinded to the animal groups conducted behavioral assessments of the mice during the light phase of the light-dark cycle. Before any testing, the mice could acclimate to the room for 30 min. All behavioral equipment was cleaned with 70% isopropanol between trials. To reduce variability, behavioral testing was performed by the same researcher at the same time each day.

### Rotarod test

The rotarod test is a method employed to assess sensorimotor function in mice [52]. For this evaluation, we used the Rotamex 5 system from Columbus Instruments (Columbus, OH) and followed the protocol as we have previously described [35, 53]. Each mouse underwent a training regimen over 2 days, with 3 daily trials, allowing them to explore the testing apparatus for a few minutes before actual testing began. The procedure started by placing the mouse on a stationary rod, allowing the animal to examine for 30 s. Then, the rod began to rotate, with the drum’s speed gradually increasing from 4 to 40 rpm. Each trial concluded when the mouse fell off the rotarod, or a 5-min period had elapsed, and the latency to fall was recorded. The mean value of these measurements served as the baseline assessment for each mouse. The testing sessions were conducted at 1, 3, 7, 14, and 35 dpi.

### Elevated plus maze test

The Elevated Plus Maze (EPM) test is a neurobehavioral paradigm that examines the conflict between a mouse’s innate exploratory drive and its fear of open areas. The EPM is designed to measure anxiety-related behaviors. It consists of a plus-shaped apparatus with 2 open and 2 closed arms. Mice typically avoid open spaces due to the increased risk of predator exposure. Therefore, animals that spend more time in the open arms are considered to exhibit less anxiety-like behavior. In our study, we utilized an EPM apparatus based on the design introduced by Pellow et al. [54]. The plus-shaped maze is custom-made using white acrylic material. It comprises 2 open arms (50 × 10 cm) and 2 closed arms (50 × 10 × 40 cm), positioned in such a way that the 2 arms of each type are situated opposite to each other and connected by a central platform (5 × 5 cm). The maze is elevated to a height of 75 cm above the floor. At 21 dpi, each mouse was gently placed at the central platform of the maze, with its head oriented toward one of the open arms. Subsequently, we recorded data pertaining to the time spent in the open arms, over a 5-min duration. These measurements were expressed as percentages.

### Forced swimming test

The Forced Swimming Test (FST) is a widely recognized animal model utilized to study despair behavior and is highly sensitive for evaluating the effects of pharmacological interventions on depression-like behaviors [55]. The FST measures the propensity of the animal to give up when placed in a cylinder filled with water from which they cannot escape. Rodents will initially try to escape, but over time, they will start to float more and struggle less, which is interpreted as a state of behavioral despair akin to depressive-like behavior. In this context, more time spent immobile (floating without trying to escape) is considered an indicator of greater depressive-like behavior. At 22 dpi, mice were placed inside glass cylinders measuring 30 cm in height and 20 cm in diameter, filled with water at a temperature of 25 ± 1 °C, reaching a depth of 15 cm. The test duration was established to be 6 min. During the final 4 min of the test, a trained observer, who was blinded to the treatment groups, recorded the behavioral responses of the mice. In this context, despair behavior in animals was quantified in terms of total swimming and immobility. Mouse immobility was passive floating with minimal movement, just enough to keep the animal’s head above the water. The cumulative time spent by the mice in this immobile state served as an indicator of behavioral despair. Additionally, any time the mice spent climbing or swimming was considered part of the total swimming time.

### Statistical analysis

Two-way ANOVA analyses were performed to evaluate the effects of sex (male vs. female) and treatment (VH vs. PP) for the 3 dpi and 35 dpi groups. For SCFA levels, two-way ANOVA was conducted, followed by Tukey’s post hoc test for pairwise comparisons. Immunofluorescence staining data, including Iba-1+, TGF-β/Iba-1+, Arg/Iba-1+, and TNF-α/Iba-1 + cells, and lesion volume (%), TUNEL + cells and microbiome analysis were evaluated using two-way ANOVA to assess the effects of sex (male vs. female) and treatment (VH vs. PP) with Tukey’s post hoc tests. For Sholl analysis, the number of intersections and average branch length were calculated separately for TBI male and female groups, with VH and PP factors analyzed using one-way ANOVA. Additionally, two-way ANOVA (sham vs. TBI, VH vs. PP) was applied to evaluate male and female groups separately for behavioral tests such as the EPM and FST, followed by Tukey’s post hoc tests. For the rotarod test, two-way ANOVA was used to analyze performance at individual time points. All mice were randomly assigned to experimental conditions, and experimenters were blinded to the treatment groups throughout the study. Statistical analyses were conducted using GraphPad Prism 8 software (GraphPad, San Diego, CA, USA). Data are presented as mean ± standard error of the mean (SEM), and significance levels were set at *p < 0.05, **p < 0.01, ***p < 0.001, and ****p < 0.0001.

## Results

### The effect of pan-probiotic treatment on gut microbiota diversity

**Fig. 2:**
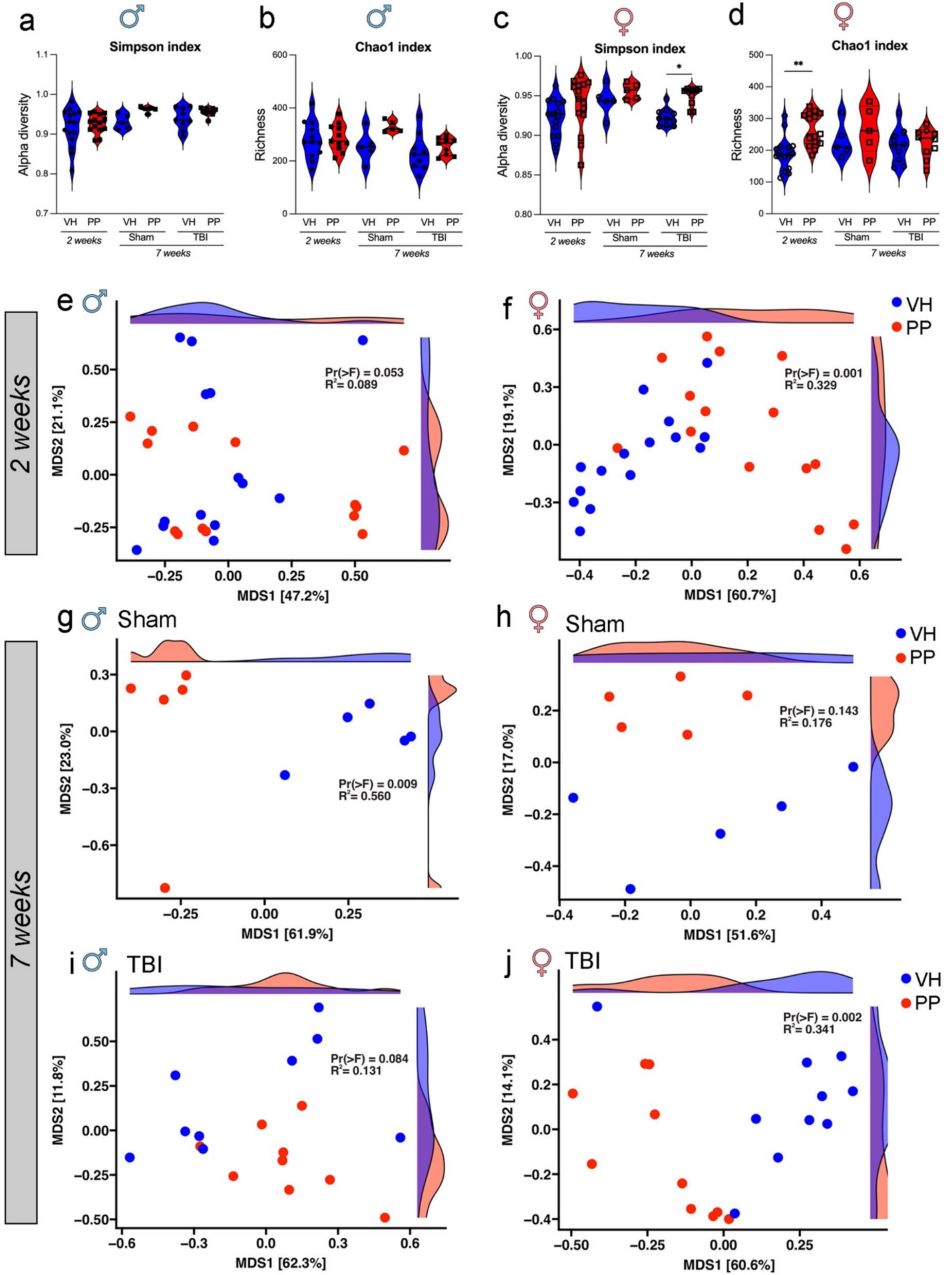
Pan-probiotic (PP) treatment influences both alpha and beta diversity in a sex-dependent manner. Alpha and beta diversity analyses of gut microbiota composition in male and female mice treated with PP or vehicle (VH) at 2- and 7-weeks post-treatment. (**a**–**d**) The Simpson index and Chao1 richness in males and females indicate that fecal microbiota diversity was higher following a 2-week PP treatment when compared to a VH treatment in both the sham (*n* = 10) and the traumatic brain injury (TBI) groups (*n* = 29–30). This pattern persisted into the 7-week treatment for both the sham (*n* = 10) and TBI (*n* = 19) groups under VH or PP treatments. Statistical significance was assessed using two-way ANOVA with post-hoc Tukey’s multiple comparison test (*p < 0.033, **p < 0.002, ***p < 0.001). (**e**–**j**) Principal Coordinate Analysis (PCoA) ordination plots were based on Weighted UniFrac distances, and PERMANOVA assessed group dissimilarities. Beta diversity showed significant differences between the female VH and PP groups after 2 weeks of treatment (**f**), the male mice in the 7-week sham group (**g**), and the female TBI PP mice after 7 weeks of treatment (**j**). Statistical significance was assessed using Tukey’s multiple comparisons test (*p<0.033, **p< 0.002, ***p< 0.001)

We evaluated whether PP treatment could affect the fecal microbiota composition and abundance based on the number of OTUs in the microbiome. In males, no significant differences were observed in alpha diversity between VH and PP groups at either 2 weeks or 7 weeks, measured by the Simpson index [56] and Chao1 richness [51] (Fig. 2a, b). A statistically significant increase was observed between the female TBI PP versus VH treatment groups after 7 weeks, as shown by the Simpson index (*p* < 0.05) (Fig. 2c). In females, a significant increase in microbial richness (Chao1 index) was observed in the PP-treated group compared to VH at 2 weeks (*p* < 0.01). Still, no significant differences were detected at 7 weeks (Fig. 2d). PP treatment resulted in significant changes in beta diversity, particularly in females at 2 weeks and 7 weeks post-treatment. In males, no significant differences in beta diversity were observed between VH and PP groups (Pr(>F) = 0.053, R² = 0.089) (Fig. 2e). In females, a significant difference in beta diversity was found between VH and PP groups (Pr(>F) = 0.001, R² = 0.329), indicating that PP treatment influenced microbial composition at 2 weeks (Fig. 2f). In males, significant differences in beta diversity were observed between VH and PP sham groups (Pr(>F) = 0.009, R² = 0.560) (Fig. 2g). In females (h), no significant differences were observed between VH and PP sham groups (Pr(>F) = 0.143, R² = 0.176) (Fig. 2h). In males, no significant differences in beta diversity were detected between VH and PP TBI groups (Pr(>F) = 0.084, R² = 0.131) (Fig. 2i). In females (j), a significant difference in beta diversity were observed between VH and PP groups (Pr(>F) = 0.002, R² = 0.341) (Fig. 2j). These findings suggest a sex- and time-dependent gut microbiome modulation by PP treatment.

### The effect of pan-probiotic treatment on gut microbiota diversity

**Fig. 3:**
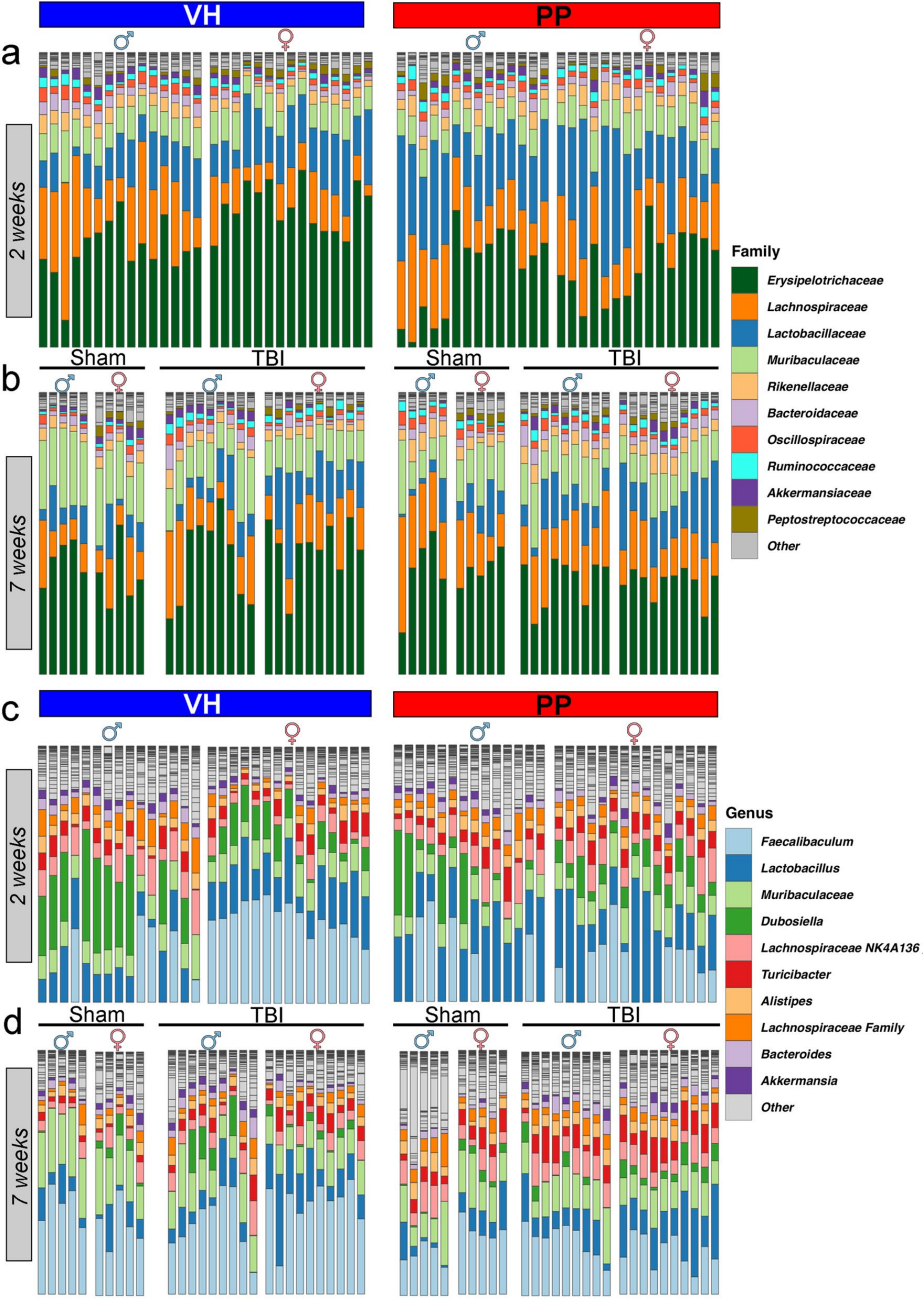
Composition of gut microbiota at the family and genus levels in male and female mice treated with Pan-Probiotic (PP) or vehicle (VH) under sham and TBI conditions. Variations in microbial composition between treatment groups, sexes, and time points are observed. (**a**, **b**) Stacked bar plots representing the relative abundance of bacterial families in male (♂) and female (♀) mice treated with VH (blue) or PP (red) at (**a**) 2 weeks and (**b**) 7 weeks post-treatment under sham and TBI conditions. Prominent families include *Erysipelotrichaceae*, *Lachnospiraceae*, *Lactobacillaceae*, *Muribaculaceae*, and *Rikenellaceae*. (**c**, **d**) Stacked bar plots showing the relative abundance of bacterial genera in male (♂) and female (♀) mice at (**c**) 2 weeks and (**d**) 7 weeks post-treatment under sham and TBI conditions. Key genera include *Faecalibaculum*, *Lactobacillus*, *Muribaculaceae*, *Dubosiella*, *Lachnospiraceae NKA4136*, and *Akkermansia*. *n* = 10 mice/ sham groups and *n* = 19–20 mice/ TBI groups

To gain a broader perspective on microbial shifts during PP treatment, family-level analysis highlighted a pronounced difference between treatment groups, primarily driven by the *Lactobacillaceae* family (Fig. 3a). At the 7-week time point, family-level microbiome analysis showed no significant alterations throughout the CCI and PP treatment, suggesting that a more detailed taxonomic resolution is necessary to fully capture the complex microbial interactions (Fig. 3b). After 2 weeks of treatment, the relative abundance of the top 10 microbial genera in VH-treated mice displayed a distinct sex-dependent pattern. In male mice, there was a pronounced presence of *Dubosiella*, contrasting with the female mice, which showed a higher abundance of *Faecalibaculum*. In the PP-treated groups, sham male mice demonstrated a higher frequency of *Lactobacillus*, while an increased presence of *Faecalibaculum* characterized their female counterparts. In TBI-PP mice, a notable dominance of *Dubosiella* was observed in males, many of which also harbored *Faecalibaculum*.

In contrast, female mice predominantly displayed an abundance of *Faecalibaculum* (Fig. 3c). By 7 weeks of treatment, there was a notable balance in the microbial composition between male and female VH-treated mice, regardless of whether they were subjected to sham or TBI interventions. Conversely, in the PP-treated groups, sex-dependent variations were more pronounced. Notable shifts in the relative abundance of *Lactobacillaceae* and *Akkermansia* highlight the impact of PP treatment on microbial diversity and its potential role in modulating gut-brain axis dynamics post-TBI. TBI-exposed female mice, however, showed a higher abundance of *Lactobacillus* relative to their male counterparts, underscoring the ongoing sex-specific influence of PP treatment in the context of TBI *(*Fig. 3d).

### The effect of pan-probiotic treatment on gut microbiota composition

**Fig. 4:**
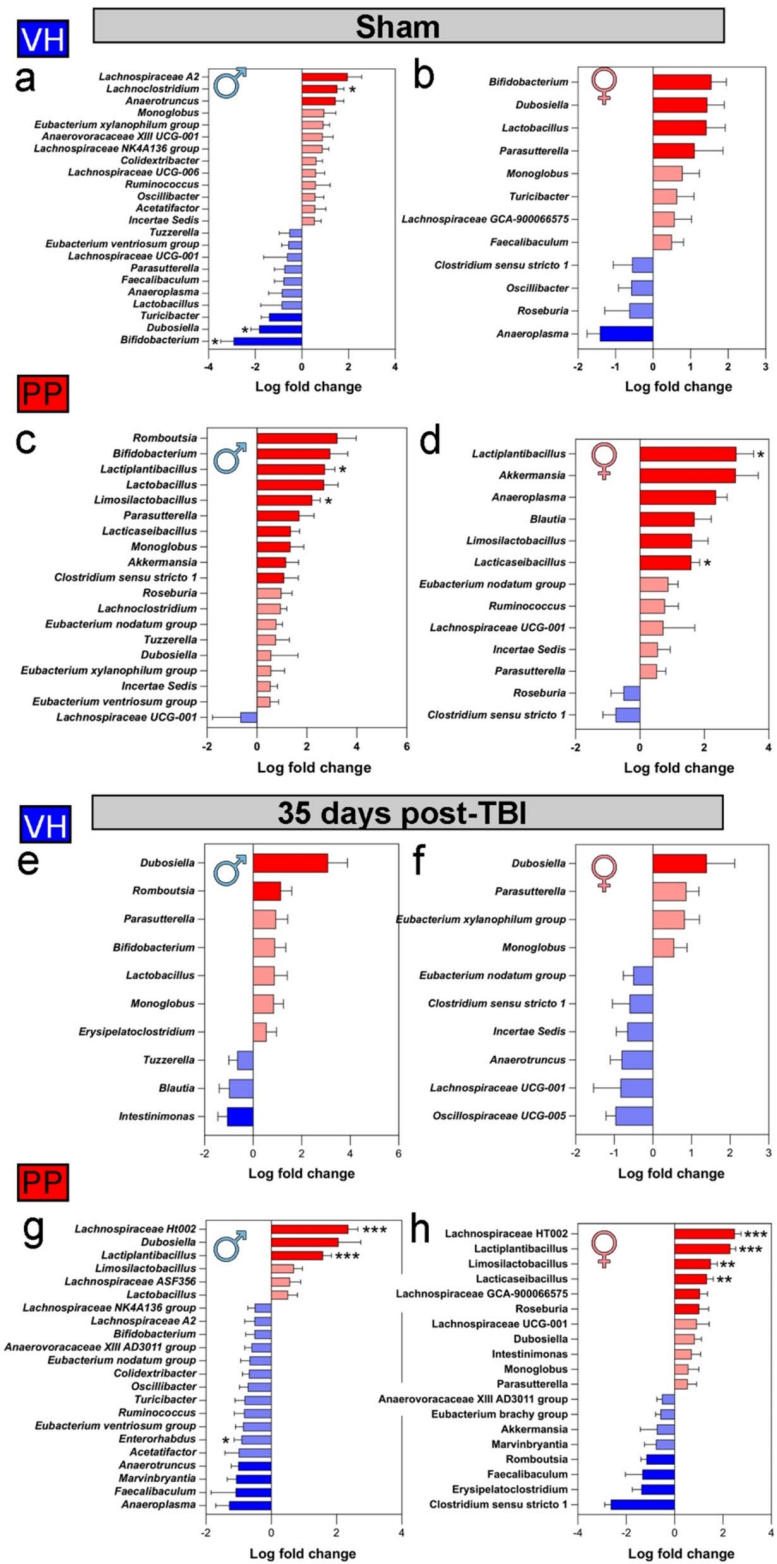
Pan-Probiotic (PP) treatment induces significant shifts in the relative abundance of specific bacterial taxa in a sex- and condition-dependent manner. The shifts in microbial communities following sham and TBI interventions, VH or PP-treated between the 2-week and 7-week time frames. The mean log fold change in abundance of a particular taxon, with the blue bars indicating a decrease and the red bars indicating an increase in abundance after 7 weeks of treatment compared to the 2 weeks of treatment. (**a**) Differential abundance analysis of microbial taxa in sham mice treated with VH revealed significant changes in males, including decreases in *Bifidobacterium* and *Dubosiella* and an increase in *Lachnoclostridium*. In contrast, no significant differences were observed in females treated with VH (**b**). In sham-PP treated groups, males (**c**) showed a notable increase in *Lactiplantibacillus* and *Limosilactobacillus*, while females (**d**) exhibited significant increases in *Lactiplantibacillus* and *Lactocaseibacillus*. In TBI groups, neither males (**e**) nor females (**f**) in the VH group showed significant changes in microbial taxa at 35 days post-injury (dpi). In contrast, males (**g**) in the TBI-PP group exhibited a notable increase in *Lactobacillaceae HT002* and *Lactiplantibacillus*, along with a reduction in *Enterorhabdus*. Females (**h**) in the TBI-PP group displayed significant shifts, including elevated levels of *Lactobacillaceae HT002*, *Lactiplantibacillus*, *Limosilactobacillus*, and *Lactocaseibacillus*. Error bars indicate the standard error of the mean (SEM). A minimum log-fold change threshold of 0.5 was applied for visualization. Statistical significance was assessed using q-values from the ANCOMBC2 analysis, with significance thresholds set at *q < 0.05, **q < 0.01, and ***q < 0.001. *n* = 10 mice/group

PP treatment induces significant shifts in the relative abundance of specific bacterial taxa in a sex- and condition-dependent manner. Changes in microbial abundance highlight the potential of PP to modulate gut microbiota composition and support recovery post-TBI. The comparative analysis examined the variations in microbial abundance between the 2-week and 7-week intervals in mice subjected to VH and PP treatments across both sham and TBI conditions. In the sham VH-treated groups, male mice exhibited slightly significant alterations in microbial taxa, including a decrease in *Bifidobacterium* (LFC=-2.93, q = 0.024) and *Dubosiella* (LFC=-1.84, q = 0.029), as well as an increase in *Lachnoclostridum* (LFC = 1.52, q = 0.024), (Fig. 4a) suggesting slight dynamic interactions are occurring within the population regardless of treatment. No changes in females sham-VH groups (Fig. 4b). We found a shift in the sham-PP male group, leading to a significantly increased abundance of *Lactiplantibacillus* (LFC = 2.73, q = 0.006) and *Limosilactobacillus* (LFC = 2.21, q = 0.011) (Fig. 4c). The genus *Lactoplantibacillus* was significantly enriched in female sham-PP mice (LFC = 3.00, q = 0.020) (Fig. 4d). A sex-specific difference was observed, as only *Lacticaseibacillus* showed a significant increase in abundance in the female PP cohort (LFC = 1.60, q = 0.011) (Fig. 4d).

Investigating the effect of TBI in both males and females, VH-treated mice did not demonstrate any significant shifts in microbial taxa, underscoring a consistent microbial profile (Fig. 4e, f). TBI-PP treatment of male mice was shown to significantly increase *Lactiplantibacillus* (LFC = 1.96, q = 4.96e-4) and *Lactobacillaceae HT002* (LFC = 2.36, q = 5.39e-4). In parallel, a significant reduction in the species *Enterorhabdus* (LFC= -0.91, q = 0.044) was also seen in the TBI-PPtreated male mice (Fig. 4g). The TBI-PPtreated female mice underwent the most significant microbial changes with increases in the abundance of *Lactiplantibacillus* (LFC = 2.31, q = 5.79e-7), *Lactobacillaceae HT002* (LFC = 2.49, q = 2.58e-5), *Limosilactobacillus* (LFC = 1.50, q = 0.001), and *Lactocaseibacillus* (LFC = 1.34, q = 0.004) (Fig. 4k). The relationship between the gut microbiomes of the VH and PP mice at the 2-week timepoint showed no significant variation of the *Lactobacillus* genus, as indicated by the differential abundance analysis. Summarizing, in males, *Lachnospiraceae HT002* and *Lactiplantibacillus* show the largest increases following PP treatment post-TBI, while females exhibit notable enrichment in *Limosilactobacillus* and *Lactiplantibacillus*.

We examined species-level results through taxonomic classification using the DADA2 “assignSpecies” function. At 2 weeks post-treatment, no detectable matches were found for any of the tested *Lactobacillus* strains in the VH treatment group. In contrast, the PP treatment group showed the presence of *Lactobacillus gasseri (*ATCC 33323) with a high confidence score (0.91), although not an exact match. Furthermore, *Lactobacillus helveticus* (ATCC BAA-2840) was identified with both a species match and an exact match, achieving a perfect confidence score of 1 (Table 1). At 7 weeks post-PP-treatment, no *Lactobacillus* strains were detected in the VH treatment group. However, in the PP treatment group, *Lactobacillus gasseri* (ATCC 33323) remained present with a slightly higher confidence score (0.93), though still not an exact match. *Lactobacillus helveticus* (ATCC BAA-2840) was again detected with a species match and an exact match, maintaining a perfect confidence score of 1 (Table 1). These results suggest that the administration of the PP treatment explicitly supports the presence of *Lactobacillus gasseri* and *Lactobacillus helveticus*, with consistent identification over time, while the VH treatment does not support detectable levels of these strains.

**Table 1:** The probiotic treatment promotes the selective persistence of *Lactobacillus* strains in the gut microbiome over time.

PAN-probiotic strain	DADA2 species match	DADA2 species-exact match	Avg. conf
2 weeks– VH treatment			
*Lactobacillus gasseri (ATCC 33323)*	No	N/A	N/A
*Lactobacillus plantarum (ATCC BAA-793)*	No	N/A	N/A
*Lactobacillus reuteri (ATCC 23272)*	No	N/A	N/A
*Lactobacillus helveticus (ATCC BAA-2840)*	No	N/A	N/A
*Lactobacillus fermentum (ATCC 23271)*	No	N/A	N/A
*Lactobacillus rhamnosus (ATCC BAA-2836)*	No	N/A	N/A
*Lactobacillus casei (ATCC BAA-2843)*	No	N/A	N/A

2 weeks– PP treatment			
*Lactobacillus gasseri (ATCC 33323)*	**Yes**	**No**	0.91
*Lactobacillus plantarum (ATCC BAA-793)*	No	N/A	N/A
*Lactobacillus reuteri (ATCC 23272)*	No	N/A	N/A
*Lactobacillus helveticus (ATCC BAA-2840)*	**Yes**	**Yes**	1
*Lactobacillus fermentum (ATCC 23271)*	No	N/A	N/A
*Lactobacillus rhamnosus (ATCC BAA-2836)*	No	N/A	N/A
*Lactobacillus casei (ATCC BAA-2843)*	No	N/A	N/A

7 weeks– VH treatment			
*Lactobacillus gasseri (ATCC 33323)*	No	N/A	N/A
*Lactobacillus plantarum (ATCC BAA-793)*	No	N/A	N/A
*Lactobacillus reuteri (ATCC 23272)*	No	N/A	N/A
*Lactobacillus helveticus (ATCC BAA-2840)*	No	N/A	N/A
*Lactobacillus fermentum (ATCC 23271)*	No	N/A	N/A
*Lactobacillus rhamnosus (ATCC BAA-2836)*	No	N/A	N/A
*Lactobacillus casei (ATCC BAA-2843)*	No	N/A	N/A

7 weeks– PP treatment			
*Lactobacillus gasseri (ATCC 33323)*	**Yes**	**No**	0.93
*Lactobacillus plantarum (ATCC BAA-793)*	No	N/A	N/A
*Lactobacillus reuteri (ATCC 23272)*	No	N/A	N/A
*Lactobacillus helveticus (ATCC BAA-2840)*	**Yes**	**Yes**	1
*Lactobacillus fermentum (ATCC 23271)*	No	N/A	N/A
*Lactobacillus rhamnosus (ATCC BAA-2836)*	No	N/A	N/A
*Lactobacillus casei (ATCC BAA-2843)*	No	N/A	N/A

### Pan-probiotics treatment increases the relative abundance of SCFAs in serum

**Fig. 5:**
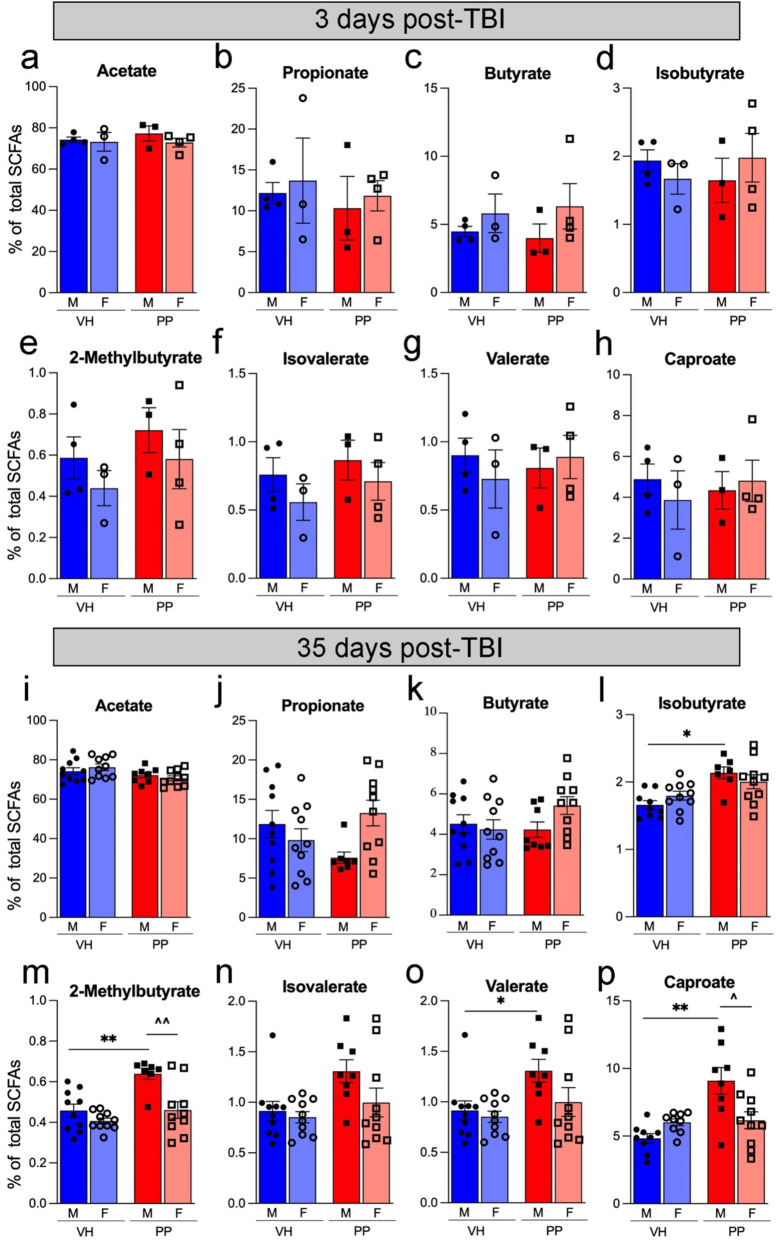
Serum short-chain fatty acid (SCFA) levels in mice treated with vehicle (VH) or Pan-Probiotic (PP) at 3 and 35 days post-TBI. Bar plots illustrate the relative abundance of individual SCFAs (as a percentage of total SCFAs) in male (M) and female (F) mice across treatment groups (VH: vehicle, blue; PP: probiotic, red). SCFAs analyzed include acetate, propionate, butyrate, isobutyrate, 2-methylbutyrate, isovalerate, valerate, and caproate. (**a**–**h**) At 3 days post-injury (dpi), no significant differences were observed among the groups. (**i**–**p**) At 35 dpi, a significant interaction effect was observed for propionate (**j**), with males showing a decrease and females showing an increase in propionate levels from VH to PP treatment. In males, PP treatment increased isobutyrate (**l**), 2-methylbutyrate (**m**), valerate (**o**), and caproate (**p**) levels in male mice, with significant differences indicated (*p < 0.05, **p < 0.01, ^*p* < 0.05 for sex comparison). Comparisons between males and females within the PP-treated group revealed that males had higher levels of 2-methylbutyrate (**m**) and caproate (**p**). Statistical significance between groups was evaluated using two-way ANOVA followed by Tukey’s post-hoc test for multiple comparisons (*p < 0.05, **p < 0.01). Sex differences were indicated by (^^*p* < 0.05). *n* = 3–4 mice/ 3 dpi group, *n* = 9–10 mice/ 35 dpi group. Error bars represent the standard error of the mean (SEM)

SCFA levels are influenced by PP treatment in a sex- and time-dependent manner, with significant increases observed primarily at 35 days post-TBI in males. At 3 dpi, there were no significant differences in the levels of acetate, propionate, butyrate, isobutyrate, 2-methyl-butyrate, valerate, and caproate between VH and PP treatment groups across both male and female mice (Fig. 5a-h). At 35 dpi, notable differences were observed; isobutyrate, 2-methyl-butyrate, valerate, and caproate levels demonstrated significant increases in the PP-treated males compared to VH, with 2-methyl-butyrate and caproate also showing significant increases in PP-treated males vs. females (**p < 0.01, *p < 0.05, respectively) (Fig. 5l-p). This suggests that PP treatment effectively replenishes specific SCFAs in the 35 dpi group after 7 weeks of treatment and that the impact of PP treatment on SCFA profiles is sex-dependent in the context of TBI, modulating both metabolic and inflammatory responses at chronic time points post-TBI.

### Pan-probiotic treatment reduces the lesions and cell death in males following TBI

**Fig. 6:**
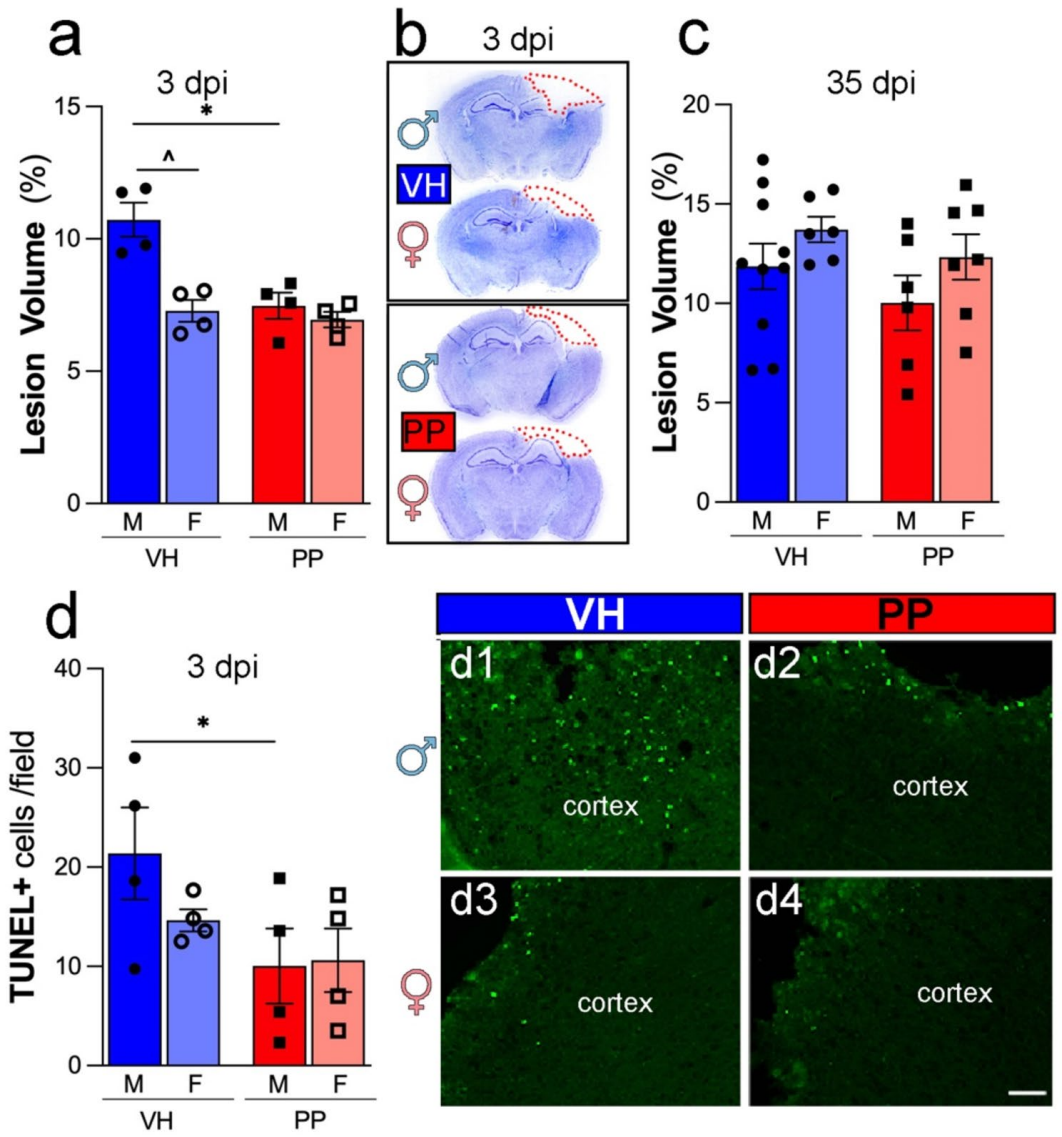
Pan-Probiotic (PP) treatment decreases lesion volume and cell death. (**a**) Lesion volume, expressed as a percentage of the total brain area, was significantly reduced in male mice treated with PP compared to VH-treated males (*p < 0.05). Female mice showed no significant differences between treatment groups, however female mice in the VH-treated group exhibited smaller lesion volumes than male mice in the same treatment group (^*p* < 0.05). (**b**) Cresyl violet-stained coronal brain sections showing lesion areas (red dashed lines) in male and female mice treated with VH or PP at 3 days post-injury (dpi). (**c**) No significant differences in lesion volume were observed between VH and PP-treated male or female mice at 35 dpi. (**d**) Male mice treated with PP showed significantly fewer TUNEL + cells compared to VH-treated males (*p < 0.05). No significant differences were observed in females. Representative fluorescent images of TUNEL + cells (green) in the cortex of male and female mice treated with VH (d1, d3) or PP (d2, d4). Statistical significance was assessed using two-way ANOVA with Tukey’s multiple comparison post-hoc test (*, ^*p* < 0.05). *n* = 4–10 mice/group. Scale bars in panels d1–d4 indicate 50 μm

PP treatment significantly reduced lesion volume and apoptotic cell death at 3 dpi in male mice, highlighting its neuroprotective effects during the acute phase of TBI. These effects were not observed in females or at the chronic time point (35 dpi). We found a significant reduction in lesion volume in male subjects treated with PP compared to VH (*p < 0.05) (Fig. 6a). In contrast, females did not exhibit a significant change at 3 dpi. Additionally, females in the VH group showed a less pronounced lesion volume than their male counterparts at 3 dpi. Representative cresyl-violet brain sections highlight the areas of the lesion (Fig. 6b). No differences in lesion volume were found in either males or females at 35 dpi (Fig. 6c). The TUNEL assay results indicated a significant decrease in dying cells in males receiving PP treatment compared to VH (*p < 0.05) (Fig. 6d, dFigs. 1, 2, 3 and 4). These findings suggest that the PP treatment may exert neuroprotective effects in a sex-specific manner post-TBI.

### Pan-probiotics treatment reduces neuroinflammatory response in males 3 days post-TBI

**Fig. 7:**
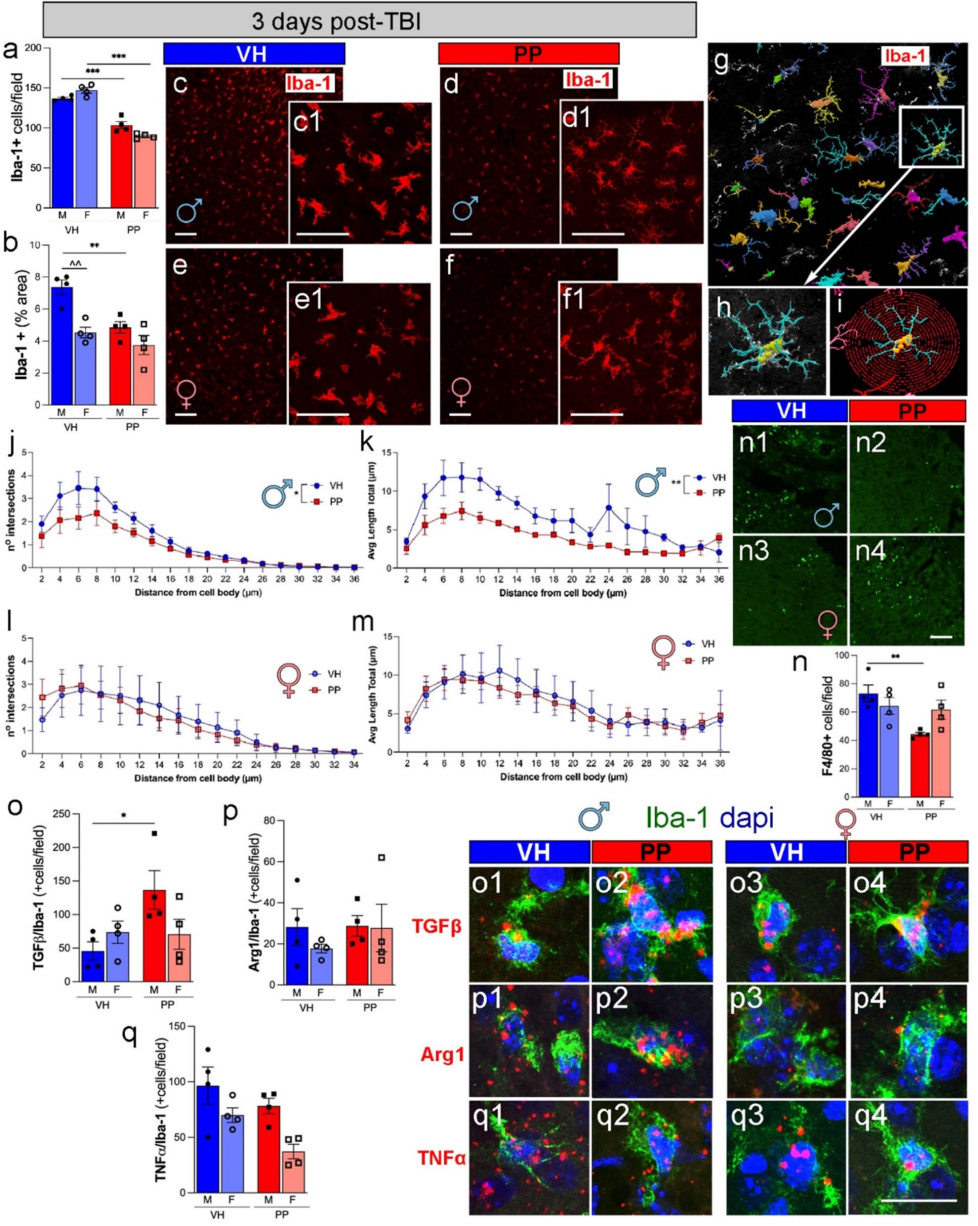
Pan-probiotic (PP) treatment decreases microglia density and macrophage infiltration at 3 days post-injury. (**a**) PP treatment significantly reduced microglial density (Iba-1 + cells, red) in male mice compared to vehicle (VH)-treated males (***p < 0.001, **p < 0.01) at 3 days post-injury (dpi). (**b**) The percentage of the area occupied by Iba-1 + cells also exhibits a noteworthy decrease in PP-treated male mice compared to their VH-treated counterparts and the VH-treated females compared with VH-treated males. (**c**, **d**, **e**, **f**) Representative images depict Iba-1 + cells in the injured cortex of mice treated with VH and PP. (**g**) Skeletonized microglial structures with color-coded branches illustrating morphological complexity using the Neurolucida 360 tracing software. (**h**) Higher magnification of individual microglia demonstrating branch intersections and total process length. (**i**) An image displays concentric circles (red) drawn at a 2 μm distance for Sholl analysis. (**c1, d1, e1, f1**) Representative confocal microscopy images of Iba-1 + staining in PP and VH brains at 40X magnification. The Sholl analysis reveals significant differences in the number of intersections from the distance of the cell body of Iba-1 + cells (**j**) and the total average length of the branch from the distance of the cell body (**k**) in the injured cortex of males treated with PP compared to VH, indicating reduced activation and more ramified microglia in PP-treated males. No differences were observed in female mice (**l**, **m**). (**n**) PP treatment reduced macrophage infiltration (F4/80 + cells, green) in male mice (**p < 0.01) but not in females. (**n1-4**) Representative images illustrate F4/80 + cells in the injured cortex of PP- and VH-treated male and female mice. (**o–q**) Co-Localization of Iba-1 + microglia with TGFβ, Arg1, and TNFα. PP treatment increased TGFβ + microglia in males (*p < 0.05) and decreased TNFα + microglia in both sexes (*p < 0.05). No significant differences were observed in Arg1 + microglia. Iba-1 + microglia (green), DAPI (blue), and markers TGFβ (**o1–o4**), Arg1 (**p1–p4**), and TNFα (**q1–q4**) in the cortex of male (**o1–q2**) and female (**o3–q4**) mice treated with VH or PP. The data represents *n* = 4 mice per group, and statistical significance was determined using two-way ANOVA with post-hoc Tukey’s multiple comparison test. For the Sholl analysis, the area under the curve was calculated, and an unpaired t-test using the mean, SEM, and n was utilized (*p < 0.05, **p < 0.01, ***p < 0.001). mRNA expression in red, and nuclei in blue. Scale bars represent 50 μm in c-f and 20 μm in c1, d1, e1, f1, o1-4, p1-4, and q1-4

To evaluate the effects of PP treatment on anti-inflammatory activity after acute TBI, we analyzed microglia and macrophage activation using the Iba-1 marker. For this quantitative analysis, our attention was directed toward the primary somatosensory cortex area proximate to the injury core. The quantitative analysis of Iba-1 + cells revealed a significant decrease in cell density in male and female mice treated with PP compared to the VH group at 3 dpi (***p < 0.001) (Fig. 7a, c-f). The Iba-1 + area within the observed fields also showed an increase, with the male VH group demonstrating a significant increase in comparison to the male PP group (**p < 0.01) (Fig. 7b, c-f). High-magnification images of Iba-1 + cells (**Fig. 7c1**,** d1**,** e1**,** f1**) revealed morphological differences, with VH-treated microglia exhibiting a more activated phenotype. No differences were observed between the contralateral hemispheres of brains treated with VH and PP (data not shown). The Sholl analysis was performed (Fig. 7g, h, i**). ** Data indicated a reduced complexity in the dendritic architecture in PP-treated males (Fig. 7g**)**, as shown by the decreased number of intersections (Fig. 7j) and the shorter average length of processes (Fig. 7k) at distances greater than 10 μm from the cell body compared to the VH-treated mice. No differences were observed in female mice (Fig. 7l, m**).** In addition, F4/80 + macrophages analysis revealed a significant elevation in cell density in VH-treated males in comparison to PP male mice (**p < 0.01) (Fig. 7n), aligning with the observed alterations in microglia morphology and suggesting a potential modulatory effect of PP on microglia activation. The females were not statistically significant when comparing PP and VH treatments. Representative images of F4/80 + staining corroborate the quantified data, with a noticeable reduction in signal intensity in the PP-treated groups compared to the VH-treated groups (Fig. 7n**1-2**). Overall, the findings suggest that post-TBI microglial activation and morphological changes are reduced by PP treatment, with more pronounced effects observed in males.

We evaluated the effect of PP on the acute anti-inflammatory response after TBI, examining the release of inflammatory cytokines at 3 dpi. No detectable mRNA expression was detected in the contralateral hemisphere or sham brains (data not shown). TGFβ, Arg1, and TNF-α mRNA expression combined with Iba-1 antibody using FISH demonstrated that most cytokine signals colocalized with microglia/macrophage markers. We quantified the positive cells for Iba-1+ cells expressing the anti-inflammatory expression of TGFβ, Arg1 mRNA, or the pro-inflammatory cytokine TNF-α mRNA in injured brain sections. We found that PP-treated males showed an increase in Iba-1+ cells expressing TGF-β compared to VH-TBI at 3 dpi, while no differences were observed in females (Fig. 7). For Iba-1+ cells expressing Arg1, no changes were detected after PP treatment in either males or females (Fig. 7p, pFigs. 1, 2, 3 and 4). We observed a trend towards reduced Iba-1/TNF-α expression in the ipsilateral peri-contusion region in PP-treated males and females compared to VH-treated mice (Fig. 7q, qFigs. 1, 2, 3 and 4). Serum amyloid A (SAA), a systemic inflammatory marker, showed no significant differences between groups. However, PP treatment exhibited a trend toward reduced SAA levels compared to VH groups, suggesting that PP treatment may attenuate the acute-phase response at 3 dpi, particularly in males (**Supplemental Fig. 1a**). Additionally, body weight measurements over 35 days revealed no significant differences between groups, with all mice displaying a typical growth curve irrespective of sex or injury (**Supplemental Fig. 1b**).

### Pan-probiotics treatment reduces neuroinflammatory response in males 35 days post-injury

**Fig. 8:**
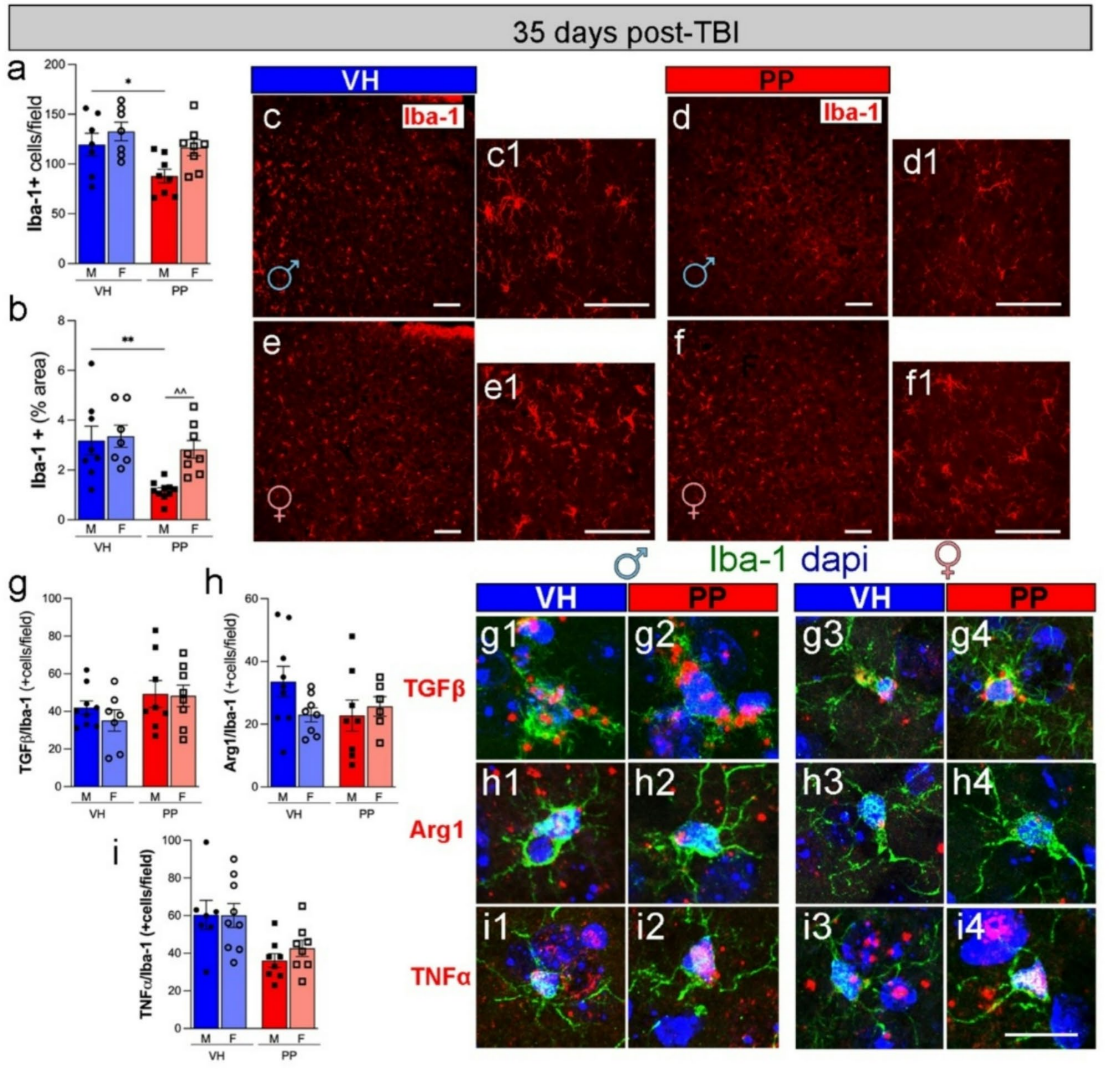
Pan-probiotic (PP) treatment decreases microglial activation and cytokine expression in male mice at 35 days post-TBI. (**a**) PP treatment significantly reduced microglial density (Iba-1 + cells, red) (*p < 0.05) and activation (**p < 0.01) in male mice compared to vehicle (VH)-treated males at 35 days post-injury (dpi). In females, a modest reduction in microglial activation was observed with PP treatment. (**b**) The percentage of the area occupied by Iba-1 + cells also exhibit a noteworthy decrease in PP-treated male mice compared to their VH-treated males. There was also a significant increase in PP-treated female mice compared to their male counterparts (**c**, **c1, d1, e1, f1**). Representative images depict Iba-1 + cells in the injured cortex of mice treated with VH and PP. Iba-1 + cells colocalizing with TGFβ + cells (**g**, **g1-4**), with Arg1 + cells (**h**, **h1-4**), and with TNF-α + cells (**i**, **i1-4**) did not show any differences after PP treatment at 35 dpi. The data represents *n* = 8–10 mice per group, and statistical significance was determined using two-way ANOVA with post-hoc Tukey’s multiple comparison test. (*p < 0.05). *n* = 7–10 mice/group. mRNA expression in red, microglia/macrophage in green, and nuclei in blue. Scale bars represent 50 μm in c-f and 20 μm in c1, e1, d1, f1, and g1-4, h1-4, i1-4

The quantitative analysis of Iba-1 + cells revealed a significant decrease in cell density in male mice treated with PP compared to the VH group at 35 dpi (*p < 0.05) (Fig. 8a, c-f). The Iba-1 + area within the observed fields also showed an increase, with the male VH group demonstrating a significant enlargement in comparison to the male PP group (**p < 0.01), and a considerable enlargement was observed in the female PP group in contrast to the male PP group (Fig. 8b, c-f). High-magnification images of Iba-1 + cells (Fig. 8c1, d1, e1 f1) with VH-treated microglia exhibiting a more activated phenotype at 35 dpi. We evaluated the effect of PP on the acute anti-inflammatory response after TBI, examining the release of inflammatory cytokines at 35 dpi. No detectable mRNA expression was detected in the contralateral hemisphere or sham brains (data not shown). No changes were detected after PP treatment in males or females for Iba-1+ cells expressing TGFβ, Arg1, and TNF-α (Fig. 8g, h, i, g1-4, h1-4, i1-4).

### Pan-probiotic treatment enhances motor function and depressive-like behavior following TBI

**Fig. 9:**
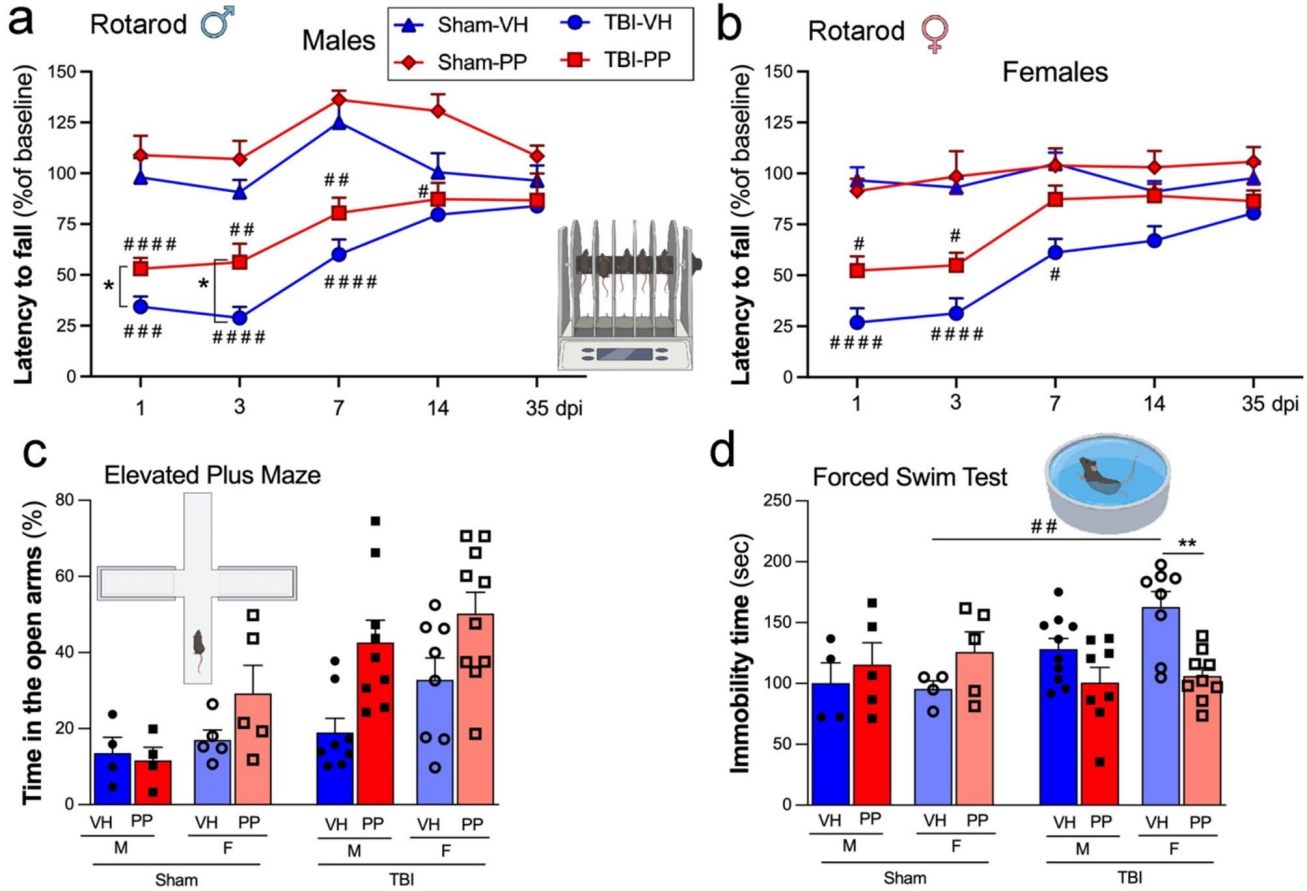
Effects of Pan-probiotic (PP) treatment on motor coordination, anxiety-like behavior, and depressive-like behavior following traumatic brain injury (TBI). Performance on the rotarod test was evaluated for both male (**a**) and female (**b**) mice, which were administered either PP or a vehicle water solution (VH). PP treatment improved motor coordination in TBI mice of both males and females compared to TBI-VH mice, with significant differences observed at various time points. Sham-PP mice also showed better performance compared to sham-VH controls. (**c**) Anxiety-like behavior was assessed using the elevated plus maze conducted at 21 dpi. TBI-PP-treated male and female mice exhibited no significant differences in the percentage time in the open arms compared to their TBI-VH counterparts. (**d**) Depressive-like behavior was evaluated using the forced swim test. TBI-VH female mice exhibited significantly increased immobility time compared tosham-VH female controls, consistent with depressive-like behavior. PP treatment significantly reduced immobility time in females compared to TBI-VH mice, suggesting an antidepressant-like effect. Data are presented as mean ± SEM. Statistical significance was determined using two-way ANOVA followed by Tukey’s multiple comparison test. *n* = 4–10 mice/group (*p < 0.05, **p < 0.01, ^#^*p* < 0.05, ^##^*p* < 0.01, ^###^*p* < 0.001, ^####^*p* < 0.0001)

In males, TBI significantly reduced latency to fall on the rotarod in VH-treated mice compared to sham-VH controls at 1, 3, and 7 dpi (^###^*p* < 0.001, ^####^*p* < 0.0001) with gradual recovery over 35 dpi, indicating a motor deficit as previously established in the CCI model [35, 39, 53, 57]. PP treatment improved motor performance in TBI male mice compared to TBI-VH at 3 and 7 dpi (*p < 0.05) (Fig. 9a). Sham groups, PP and VH treated, exhibiting reduced latency to fall compared to TBI groups in females (^####^*p* < 0.0001 at 1 and 3 dpi, ^#^*p* < 0.01 at 7 dpi), however the PP treatment did not show improvements (Fig. 9b).

In the EPM, both male and female sham and TBI treated with PP show no significant difference in the time in the open arms entries compared to VH groups (Fig. 9c). In the FST, PP treatment significantly reduced immobility time in TBI females compared to TBI-VH female mice, suggesting an antidepressant-like effect of PP treatment (**p < 0.01) (Fig. 9d). It was also observed that females in the VH-injured group exhibited more immobility than females in the sham-VH group (^##^*p* < 0.01). However, these differences were not observed in animals treated with PP. In both sham and TBI groups, male PP-treated mice did not show significant differences in immobility time compared to VH-treated mice, although a trend toward decreased immobility was noted.

## Discussion

The preventive administration of probiotics capable of modulating gut microbiota and influencing post-TBI inflammatory responses holds significant clinical relevance, particularly for active military personnel and contact athletes with a heightened risk of brain injury [58]. In this study, we administered a PP cocktail via drinking water for 7 weeks to promote intestinal colonization and assess its effects in sham mice and at 3 and 35 days post-TBI. To the best of our knowledge, this is the first study to show that a cokctail of PP with *Lactobacillus* strains modifies gut microbiome diversity and composition in a sex-dependent manner while simultaneously influencing both acute and chronic neuroinflammatory responses following TBI.

The temporal dynamics of microbial communities provides valuable insights into the sex-specific effects of PP treatment in the context of TBI. Our findings demonstrate that gut microbiota dysbiosis induced by TBI can be reversed through PP administration, which concurrently exerts neuroprotective effects on the injured brain. This builds on our previous work, which highlighted the impact of TBI on bacterial dysbiosis, particularly in the relative abundance and diversity of the *Lactobacillaceae* family [19], a group of bacteria commonly associated with a healthy gut microbiome [26]. The genus *Lactobacillus*, belonging to the phylum *Firmicutes* [59], has been well-documented for its neuroprotective properties [60]. Specifically, our earlier study identified a significant reduction in *Lactobacillus gasseri* 24 h post-TBI [19]. Furthermore, we demonstrated that probiotics alleviate cognitive and metabolic deficits associated with TBI by modulating gut microbiota composition and altering hepatic lipid profiles [61]. These findings establish a foundation for exploring probiotics as a potential therapeutic strategy for mitigating the adverse effects of TBI. In this study, we administered a probiotic mixture containing multiple beneficial *Lactobacillus* strains, including *L. plantarum*, *L. reuteri*, *L. helveticus*, *L. fermentum*, *L. rhamnosus*, *L. gasseri*, and *L. casei*. The selection of these strains was based on existing literature highlighting their beneficial effects in various animal models, particularly their role in modulating inflammatory responses, a critical aspect of many disease processes [62–65]. Our findings demonstrate that PP administration exerts partial preventive and restorative effects during the acute and chronic phases in a mouse model of TBI. Different *Lactobacillus* strains have demonstrated the capacity to reduce the production of inflammatory cytokines such as TNF-α and IL-6 [66, 67], downregulate pro-apoptotic neuronal pathways, and modulate inflammatory mediators in mouse models [68]. Notably, the probiotic *L. acidophilus* has been shown to aid in repairing the intestinal mucosal barrier and enhancing microbial diversity in mice [69]. Multi-strain probiotics treatment can ameliorate cognitive impairment and pathological change in SAMP8 mice, including neural damage, Aβ and Tau pathology, and neuroinflammation [69]. Probiotic supplementation has been shown to reduce apoptosis and improve anti-inflammatory states in a mouse model of stroke [70]. In the context of clinical TBI, *Lactobacillus* administration has been associated with reduced locomotor dysfunction, anxiety, and depression in patients [71]. Furthermore, a probiotic regimen comprising *Bifidobacterium*, *Lactobacillus*, and *Enterococcus faecalis* significantly decreased pro-inflammatory cytokine levels in patients with severe TBI [72]. Although probiotics may not establish permanent colonization in the gut, studies suggest that their long-term administration can transiently alter the intestinal microbiota composition, influencing microbial dynamics and host interactions [73].

The beneficial effects of probiotics are largely attributed to their metabolite profiles, particularly SCFAs like acetate, propionate, and butyrate [74], which promote neuroprotection [75], neuronal survival, plasticity [76], and anti-inflammatory properties [30] while modulating microglial activation [77], in both central and peripheral systems, regardless of their ability to establish long-term intestinal colonization [68, 73]. Our findings demonstrate that a 7-week PP treatment increases the relative abundance of SCFAs in the serum of mice at 35 days post-TBI, while no such increase was observed at 3 days post-TBI. This temporal discrepancy suggests that the acute inflammatory response following TBI may suppress the immediate release of SCFAs into the bloodstream, potentially due to gut dysbiosis and impaired microbial metabolism during the acute phase [19]. Despite this, the treatment appears to confer neuroprotective effects in the injured brains during the acute phase by alternative mechanisms. By 35 days post-TBI, the observed elevation of SCFAs in PP-treated groups indicates a recovery of microbial activity and underscores the potential role of SCFAs in promoting TBI recovery. These findings support the hypothesis that SCFAs contribute to resolving neuroinflammation and enhancing neuroprotection, highlighting their critical role as mediators of the gut-brain axis during the recovery phase. Sex-based differences in 2-methylbutyrate and caproate levels were observed in males, aligningwith previous studies reporting sex-related variations in SCFA profiles. However, no such differences were observed in the more abundant SCFAs (acetate, propionate, and butyrate) which are commonly studied and have previously been shown to remain unaffected by sex [78, 79].

There were increases in microbial counts and significant variations. Our findings reveal that the substantial rise in Simpson diversity observed in the PP-treated TBI group at 35 dpi in females is likely due to an elevated microbial load in the gut induced by the PP treatment. This treatment also increased richness in the PP group in females after 2 weeks. Our results also demonstrated a more pronounced beneficial impact of probiotics in males than females, align with an increasing body of evidence suggesting that male and female brains may exhibit distinct responses to TBI and subsequent treatments [37, 39, 57]. These analyses underscore sex-dependent differences in responses to PP treatment, suggesting that the gut microbiota may affect circulating hormone levels and neurotransmitter availability by modulating reabsorption rates [80]. Additionally, inherent differences in gut microbiota profiles between males and females [81] likely influence how probiotic strains colonize and interact with the host gut microbiome.

We observed specific shifts in microbial taxa in PP-treated mice, including increased abundances of beneficial bacteria such as *Lactobacillaceae*, *Limosilactobacillus*, and *Lactiplantibacillus*. Previous studies have linked *Limosilactobacillus* and *Lactiplantibacillus* to anti-inflammatory properties and improved gut health [82]. Notably, the TBI-PP group exhibited more complex taxonomic changes following TBI, with significant increases in taxa such as *Lachnospiraceae HT002*, *Lactiplantibacillus*, *Limosilactobacillus*,* and Lacticaseibacillus*, suggesting these specific taxa may play a role in promoting recovery or mitigating damage in the context of TBI as previous reports have demonstrated [71, 83–85]. These findings highlight the role of specific microbial taxa in modulating the gut-brain axis and contributing to recovery post-TBI.

However, a limitation of our current study is that the characterization of gut dysbiosis through short-read 16S sequencing is constrained by the small amplicon size, which may lead to potential taxonomic misidentification [86]. 16S amplicon sequencing is much more affordable and scalable than shotgun metagenomics. However, the inability to broadly classify bacteria at the species and strain levels and the lack of accurate functional annotations that can be performed are significant detriments when attempting to understand the gut microbiome changes [87]. To gain a broader understanding of the changes in the *Lactobacillaceae* family following PP treatment, we employed a best practice approach for short read 16S species-level identification using DADA2 [86, 88, 89]. One promising area that spans the gap between short-read amplicon sequencing and shotgun metagenomics is nanopore full-length amplicon sequencing, which has been gaining popularity in microbiome research through its ability to identify bacteria taxa at the species level [90], which we will implement in future studies.

Our findings indicate that PP treatment may enhance brain resilience to TBI by modulating systemic inflammation and fostering a neuroprotective environment prior to injury. Furthermore, PP appears to support repair mechanisms and reduce secondary injury processes, including neuroinflammation and neurodegeneration, following TBI. However, a limitation of this study is the inability to differentiate the effects of PP administration before versus after TBI. Future research will aim to clarify these temporal dynamics and provide a more detailed understanding of PP’s therapeutic impact. We also found a reduction of microglia activation at both 3 and 35 days post-TBI. Specific microbial shifts observed in our study may have primed microglia, influencing their response to injury [91]. Furthermore, dietary supplementation with microbial metabolites has been shown to improve memory and cognitive functions by suppressing microglia-mediated neuroinflammation and modulating the microbiota-gut-brain axis [92]. These insights highlight the potential of PP treatment to impact both pre-injury resilience and post-injury recovery through gut-brain axis modulation.

*Lactobacillus* supplementation has been associated with reductions in anxiety and depression [93]. Research in neonatal mice has shown that *L. rhamnosus* and *L. helveticus* can mitigate stress-induced cognitive dysfunctions [94]. Additionally, a clinical study reported that a 4-week multispecies probiotic intervention significantly reduced overall cognitive reactivity to sad mood [95]. In our study, PP treatment improved motor functions and partially alleviated anxiety-related behaviors. However, larger group sizes may be necessary to achieve statistical significance.

## Conclusions

Our study demonstrates the potential of PP supplementation to modulate the gut microbiome, metabolic profiles, and inflammation while providing neuroprotective and behavioral benefits following TBI in mice. These findings highlight the promise of probiotics as therapeutic tools for clinical TBI recovery, emphasizing the importance of gut-brain axis interactions and sex-specific responses. Further research is needed to clarify the underlying mechanisms and long-term implications contributing to personalized TBI rehabilitation approaches.

## Electronic supplementary material

Below is the link to the electronic supplementary material.

Supplementary Material 1

## Data Availability

The sequencing data used in the study have been deposited to NCBI SRA under BioProject ID PRJNA1095399. The data analysis pipeline and code used to generate this study’s findings can be found in the GitHub repository (https://villapollab.github.io/pan_probiotic_tbi/).

## Abbreviations

AhR: Aryl hydrocarbon receptor
ANOVA: Analysis of variance
ASVs: Amplicon sequencing variants
BBB: Blood-brain barrier
BCL: Binary base call
BDNF: Brain-derived neurotrophic factor
CCI: Controlled cortical impact
CFU: Colony forming unit
CNS: Central nervous system
CRP: C-reactive protein
dpi: Days post-injury
EPM: Elevated Plus Maze Test
FST: Forced Swimming Test
GF: Germ-Free
HEPA: High-efficiency particulate air
MFI: Mean fluorescence intensity
MRM: Multiple reaction monitoring
MRS: De Man, Rogosa, and Sharpe agar
NGS: Normal goat serum
PBS: Phosphate-buffered saline
PBS-T: PBS enhanced with 0.5% Triton X-100
PBS-Tw: PBS-Tween 20
PCR: Polymerase chain reaction
PP: Pan-Probiotic
rpm: Revolutions per minute
SAA: Serum amyloid A
SCFAs: Short-chain fatty acids
TBI: Traumatic brain injury
TUNEL: Terminal deoxynucleotidyl transferase dUTP nick end labeling
VH: Vehicle
