# Supplementary figures and images for: Probiotic treatment induces sex-dependent neuroprotection and gut microbiome shifts after traumatic brain injury
Source: J Neuroinflammation. 2025 Apr 20;22:114. doi: 10.1186/s12974-025-03419-1 (PMC12010691; doi:10.1186/s12974-025-03419-1)

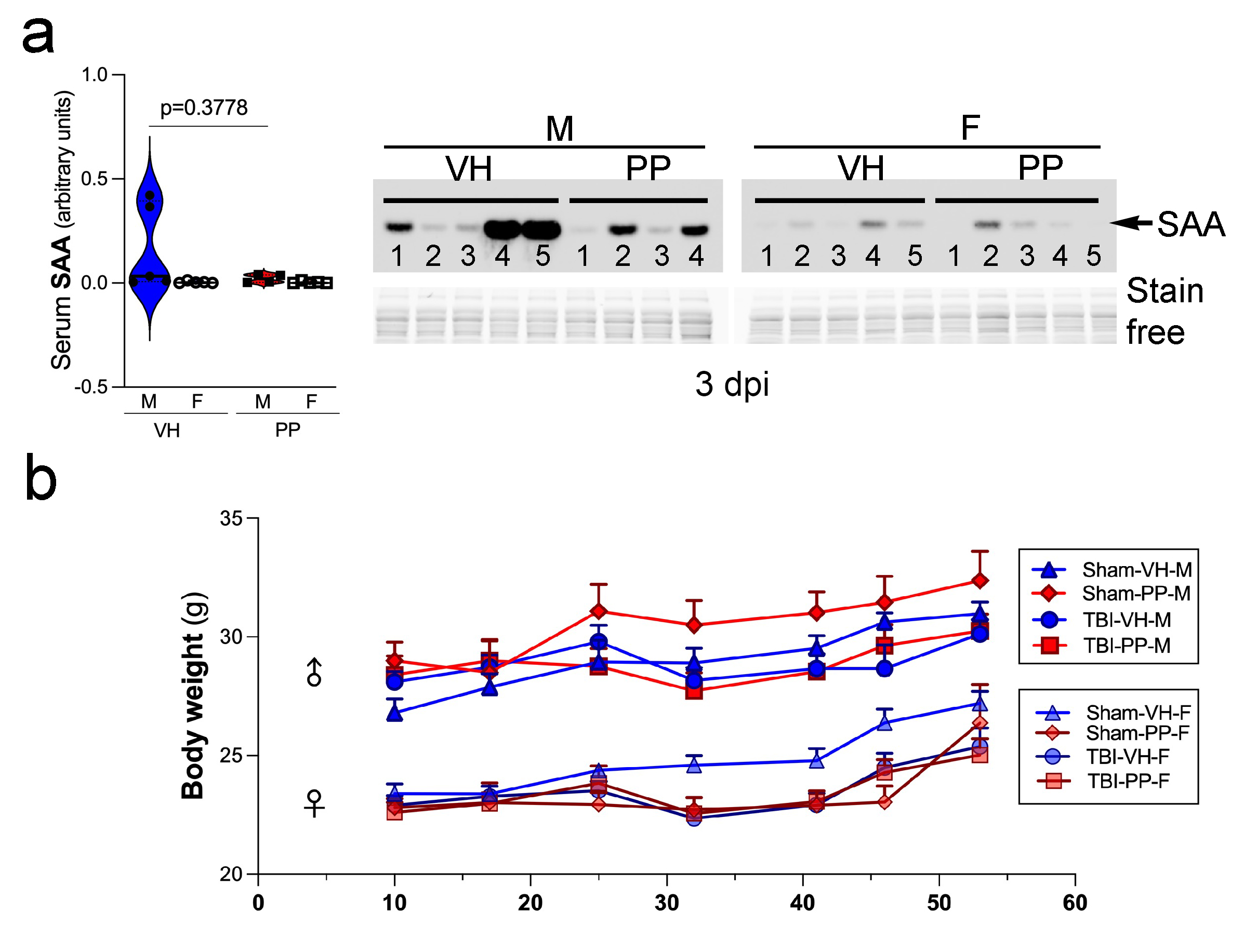

Supplement: Supplementary file 1 — Supplementary Material 1 [file 12974_2025_3419_MOESM1_ESM.jpg]
